# Tunnel failure mechanism during loading and unloading processes through physical model testing and DEM simulation

**DOI:** 10.1038/s41598-021-96206-w

**Published:** 2021-08-18

**Authors:** Yuzhou Xiang, Zhikai Zeng, Yangjun Xiang, Erdi Abi, Yingren Zheng, Hechuan Yuan

**Affiliations:** 1 Chongqing Chengtou Road and Bridge Administration, Chongqing, 400060 China; 2 National Inland Waterway Regulation Engineering Research Center, Chongqing Jiaotong University, 66 Xuefu Road, Nan’an District, Chongqing, 400074 China; 3 PLA Army Logistic University of PLA, Chongqing, 400045 China

**Keywords:** Natural hazards, Solid Earth sciences, Engineering

## Abstract

Geo-materials may present varying mechanical properties under different stress paths, especially for tunnel excavation, which is typically characterized by the decreased radial stress and increased axial stress during the complex loading and unloading process. This study carried out a comparative analysis between the loading and unloading model testing, which was then combined with PFC^2D^ simulation, aiming to reveal the fracture propagation pattern, microscopic stress and force chain distribution of the rock mass surrounding the tunnel. Comparisons of extents and development of tensile strain between loading and unloading testing results were made. The overall stability, the integrity of rock mass, and the failure pattern transition under loading and unloading processes were systematically examined. In addition, for the two unloading cases with different vertical stresses imposed, the failure patterns were both identified as the collapse of the V − shaped extruded sidewall, due to the coupling of the shear failure and the vertical tensile failure in the sidewall wedge.

## Introduction

Tunnel excavation is typically a complex loading and unloading process, accompanied with stress redistribution in the surrounding rock masses, cross-sectional convergence, and possible tunnel instability and failure, among which the latter two issues are major concerns of engineers. Therefore, it is of great importance to accurately reveal the mechanics behind the tunnel excavation process in terms of the tunnel design and construction. So far, extensive studies on relevant topics have been carried out by those literature^1–37^.

So far, extensive studies mainly focus on similar model test^1–4^, field test^5–8^, numerical analysis^9–18^ and analytical solutions^19–26^. In the similar model test research, a small tunnel model^1,2^, a transparent geotechnical engineering model^3^ and a geomechanical model^4^ mainly used to explore the passive failure mechanism of the working face of the tunnel, the distribution law of soil deformation near the tunnel, the deformation and failure characteristics of tunnels under high in-situ stress. According to field tests and data, Li et al^5^ found that vertical deformation of underground tunnels exhibits different temporal and spatial rules under rapid excavation conditions. Shreedharan^6^ concluded that longer supports and floor bolting were used to enhance the stability of a horseshoe-shaped and an inverted arch-shaped tunnel. Avgerinos^7^ discussed the impact of excavating the Crossrail tunnels under the existing Central line tunnels. Goh^8^ proposed a new assessment method combining multiple adaptive regression spline (MARS) method and logical regression (LR) method for mining excavation engineering. The undrained stability of rectangular tunnels^9^, the influence mechanism of ground support on the working face of shallow buried shield tunnel^10,11^, the stability of the surrounding rock in underground mine^12,13^, the progressive failure process of surrounding rock of jointed tunnel^14,15^, the failure behavior of a non-persistently jointed rock mass under different stress states^16^, the effects of different design parameters on twin cavern interaction^17^, the working face collapse pressure of a circular tunnel driven by a pressurized shield^18^ were studied from three dimensional kinematics, finite elements, discrete elements, RFPA and PFC3D numerical methods. Through digital image processing, the meso-crack damage and failure law of 17Mn1Si steel concluded by Maruschak et al^19,20^. Using analytical solutions, the scholars established the empirical formula of safety factor and cavern size^21,22^ and the relationships between the maximum surface subsidence^23^, calculated the lower- and upper-bound solutions for the ultimate surcharge loading of circular tunnels^24^ and the Mindlin’s solution of the unloading stress at the tunnel excavation^25^, determined the failure probability of the tunnel in the limit state^26–28^. Based on these analytical methods, so many deformation control and stability analysis approaches of surrounding rock mass have been proposed ^29–37^.

The deformation and strength properties of geo-materials are via for different stress paths, and thus these parameters will change with differences in loading and unloading conditions. Previous studies highlighted the mechanical features of the soil mass along different stress paths^38–40^. However, all the constitutive models and relevant parameters used in calculation currently are originated from the conventional experiments without consideration of the excavation − induced stress path that the soil mass has gone through. The loading stage is more commonly used to study the failure mode^3–5,41^. In such cases, the stress state and displacement field variation during the failure propagation are different from those in the actual unloading process of tunnel excavation, and hence no effective reflection upon the influence of unloading tunnel excavation on surrounding rocks can be anticipated. Given the aforementioned, the study on tunnel excavation by loading method is to some extent limited, especially when it comes to deeply understand the regularities behind the surrounding rock deformation and failure led by excavation disturbance. At present, tunnel stability analyses incorporating the excavation unloading effect are rarely seen in Li’s research^5,42–45^. To systematically analyze the tunnel stability during excavation, this paper conducts a comparative loading/unloading experiment during tunnel excavation along with the discrete element method (DEM) to shed light upon the failure plane propagation and failure mechanism during the loading and unloading processes. New insights into the tunnel deformation and failure caused by excavation were obtained, which can provide vital guidance on tunnel design and construction.

## Model testing design

The experimental prototype is a subway tunnel in Chongqing, China, with a span of 12 m and a height of 18 m. The surrounding rock is composed of IV grade sandstone and mudstone. The mechanical parameters of rock mass are selected according to *the Code for Design of Highway Tunnel*, as shown in the table. The similarity ratio between prototype and model is determined as follows: geometric similarity ratio C_*l*_ = 150, bulk density similarity ratio C_*ρ*_ = 1, Poisson's ratio similarity ratio C_*v*_ = 1, strain similarity ratio C_*ε*_ = 1, internal friction Angle similarity ratio C_*φ*_ = 1, and elastic modulus similarity ratio C_*e*_ = 150.

The model was prepared by using composite materials made up of silica sands (aggregates), gypsum, talcum powder and cement (cementing materials) mixed with a certain amount of water, in the proportion of sand, gypsum, talcum powder, cement, water = 1:0.6:0.2:0.2:0.35, by weight.

**Figure 1:**
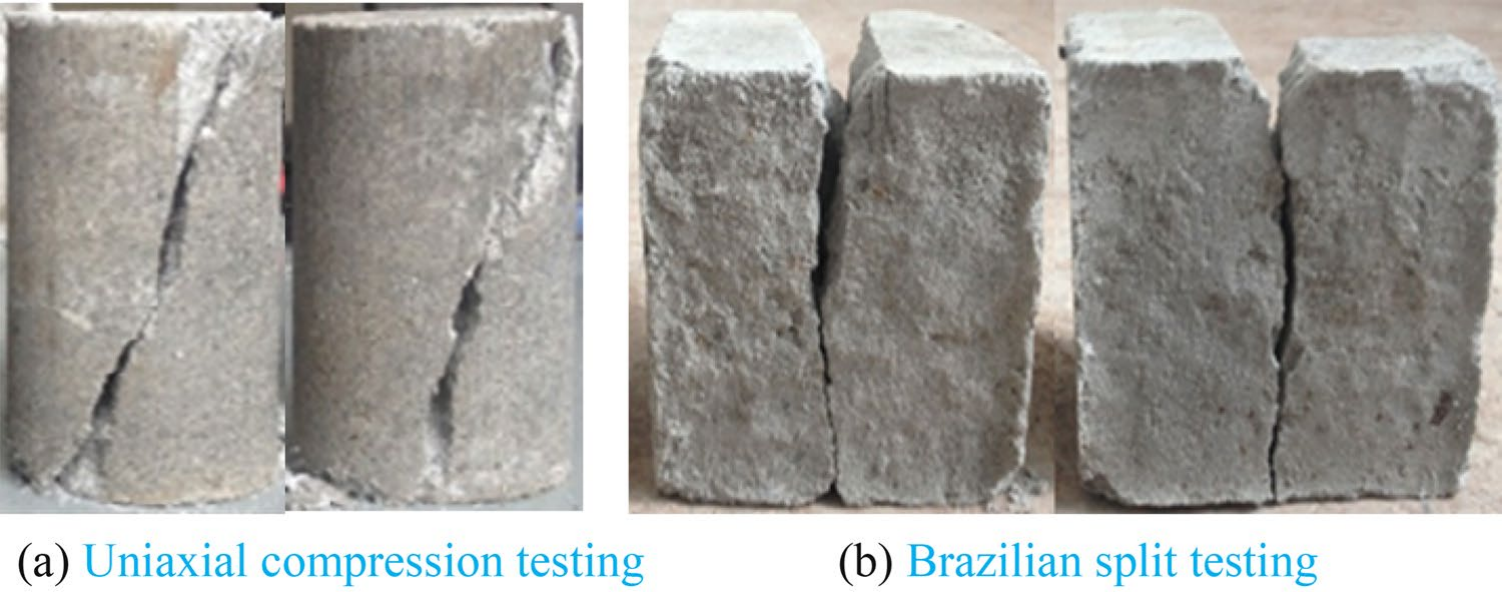
Composite material failure in T-C measurements.

Uniaxial compression testing and Brazilian split testing were carried out to determine the tension–compression (T-C) ratio of composite materials, using computer-controlled constant loading compression testing machine, as is shown in Fig. 1. Test results are concluded in Tables 1 and 2. The uniaxial compressive strength (UCS) and tensile strength of composite materials are respectively 4.628 MPa and 0.355 MPa. As the load reached the peak value during the uniaxial compressive testing, the composite materials sample broke instantly with a clear sound, which indicates notable brittleness.

**Table 1:** Results of Uniaxial compression testing.

Material	No	Peak load*/* kN	Fracture angle *f/*^o^	Specimen dimension *Ф* × *H/*mm	UCS *σ*_*cu*_*/*MPa	Average UCS *σ*_*cu*_*/*MPa
Composite material	1	5.83	72	39.2 × 80	4.831	4.628
	2	5.69	68	39.2 × 80	4.715	
	3	6.26	66	39.2 × 80	5.187	
	4	4.83	67	39.2 × 80	4.002	
	5	5.50	69	39.2 × 80	4.557	
	6	5.40	67.5	39.2 × 80	4.471

**Table 2:** Results of Brazilian split testing.

Material	No	Peak load*/*kN	Specimen dimension *a* × *b* × *H/*mm	Tensile strength *σ*_*cu*_*/*MPa	Average tensile strength *σ*_*cu*_*/*MPa
Composite material	1	7.07	100 × 100 × 100	0.450	0.355
	2	5.54	100 × 100 × 100	0.353	
	3	4.11	100 × 100 × 100	0.262	

The strength parameters of composite material were measured via laboratory tests and results are shown in Table 3.

**Table 3:** Physical mechanical parameters of the composite material.

Composite material	Elastic modulus *E/M*Pa	Poisson’s ratio, *v/*1	Unit weight, *ρ/*(kN.m^−3^)	Cohesion, *c/*MPa	Internal friction angle, *f/°*	T-C ratio
Prototype	1300 ~ 6000	0.30 ~ 0.35	27 ~ 29	0.2 ~ 0.7	27 ~ 39	13.0
Model	42.22	0.20	18	0.509	25.1	13.04

### Loading device for the testing

**Figure 2:**
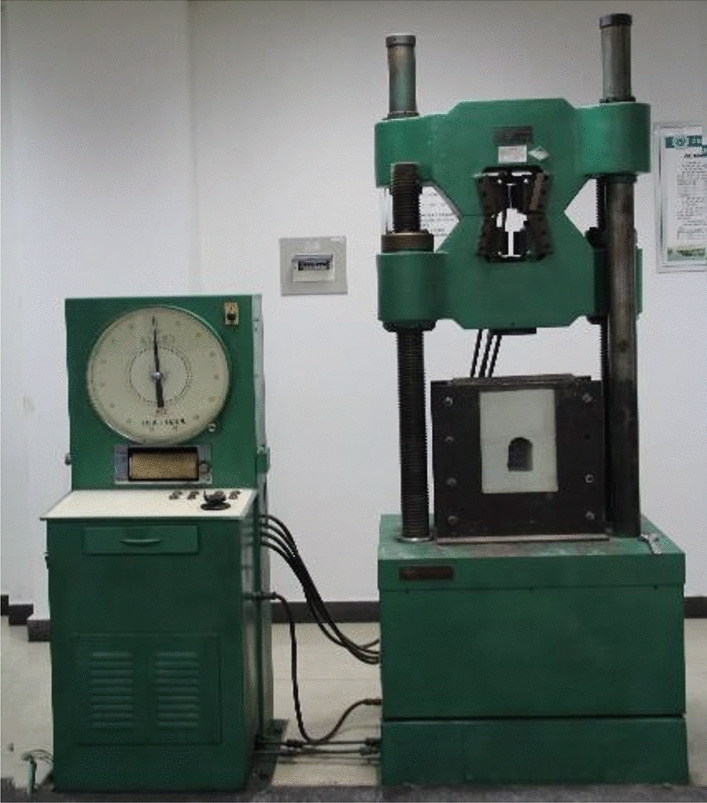
Photo of the universal testing machine.

The loading and unloading testing were accomplished by using the Model WE − 600B hydraulic universal testing machine, as shown in Fig. 2 that is competent to meet the requirements of applying load to loading and unloading models, with a maximum axial load capacity of 600 kN.

### Testing apparatus of the physical model

**Figure 3:**
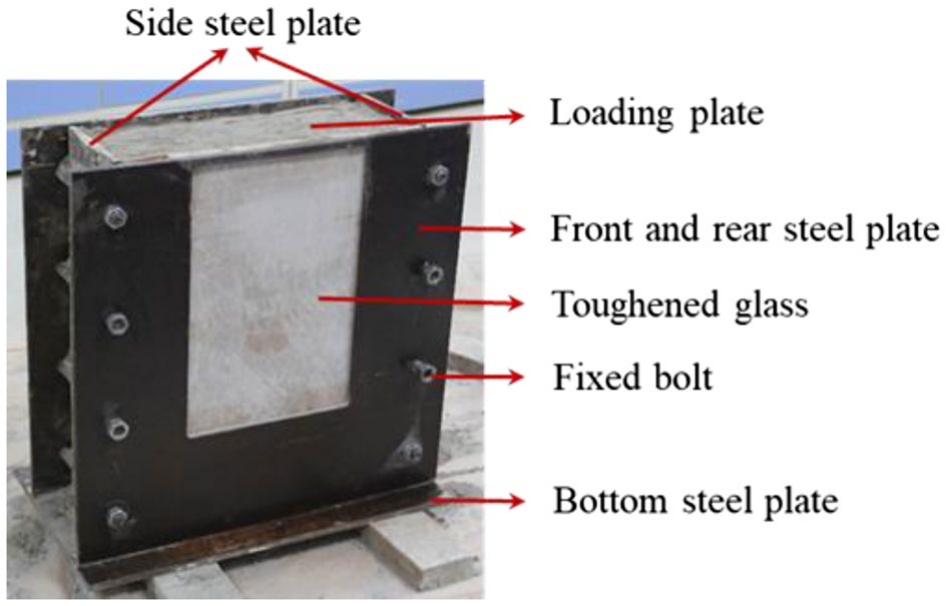
Testing model.

A corresponding tunnel model testing apparatus was designed and fabricated to simulate the tunnel failure during loading and unloading. The dimensions of the model was 56 cm × 15 cm × 52 cm (length × width × height), as shown in Fig. 3. It was mainly incorporated with the front and back steel plates with a size of 56 cm × 52 cm. A 24 cm × 30 cm steel plate on the side of observation was moved out, between which and model a piece of 2 − cm − thick tempered glass was installed so as to continuously monitor the tunnel deformation and failure. Two 15 cm × 52 cm steel plates were fixed separately on the left and right sides of the model for lateral constraints. The bottom steel plate platform was 56 cm × 25 cm. The whole model was fixed by eight bolts for better constraints. The loading steel plate, with a size of 40 cm × 3 cm × 15 cm (length × width × height), was installed on the top surface. The model was prepared by layer-by-layer manner. The tempered glass was temporarily replaced by a plank with identical dimensions during the preparation process in order to prevent from scratching or crushing then it was put back prior to the testing.

Before the physical model preparation, the steel plates were firstly fixed at the given positions by bolts. Then, silica sands, gypsum, talcum powder, cement and water were mixed thoroughly and quickly in the prescribed proportion, after which the mixture was filled into the mould layer by layer with 8-cm thick. Each layer has to be sufficiently punned, prior to packing the next layer. After 28-day curing time, the testing material reached the required strength, then strain gauges that were used to record the strain variation of the testing model during the loading and unloading processes were glued to the model surface.

### Boundary conditions of the physical model testing

The plane strain constraints were applied to the model, which meant the front, back, left and right steel plates were restrained with no displacement variation. Uniform vertical load σ_z_ was exerted on the top surface of the model, with the help of the hydraulic universal testing machine.

### Loading failure testing scheme

Prior to loading, a tunnel, with an 8 cm span, an 8 cm height vertical wall and a 4 cm arch rise, was excavated inside the physical model in advance. During the testing, information of strain variation, subsidence, fracture propagation and so on were recorded, and the peak load σ_zmax_ was identified. The first collapse of the sidewall rock was defined as the first failure. Upon the primary failure, the shape of the tunnel changed, and thus a new tunnel profile was formed. However, at this time, the model could still sustain load. As loading proceeding, the failure zone enlarged and secondary failure occurred, finally whole collapse happened. During the loading testing, loading would be stopped when penetrated failure plane or whole collapse was observed, and the load with respect to the primary failure was defined as peak load (σ_zmax_).

### Excavation unloading failure testing scheme

At first, a homogeneous physical model was prepared. The initial surrounding rock stress was applied based on the peak load of the loading testing (60% or 100% σ_zmax_). Afterwards, excavation was implemented from one side of the model, during which the deformation and failure characteristics of surrounding rock induced by the excavation disturbance were noted. In the case that the model remained stable after tunnel excavation, a loading process was followed and continued until it failed. The detailed testing program is shown in Table 4.

**Table 4:** Testing scheme design.

Scheme no	Tunnel span*/*cm	Vertical wall height*/*cm	Arch rise*/*cm	Initial surrounding rock stress $\sigma_{z}$*/*MPa	Loading pattern
1	8	8	4	0.00	First excavation then loading
2	8	8	4	60% of $\sigma_{z\;\max }$	First loading then excavation
3	8	8	4	$\sigma_{z\;\max }$	First loading then excavation

## Tunnel loading and unloading testing

### Loading failure testing

#### Fracture propagation

**Figure 4:**
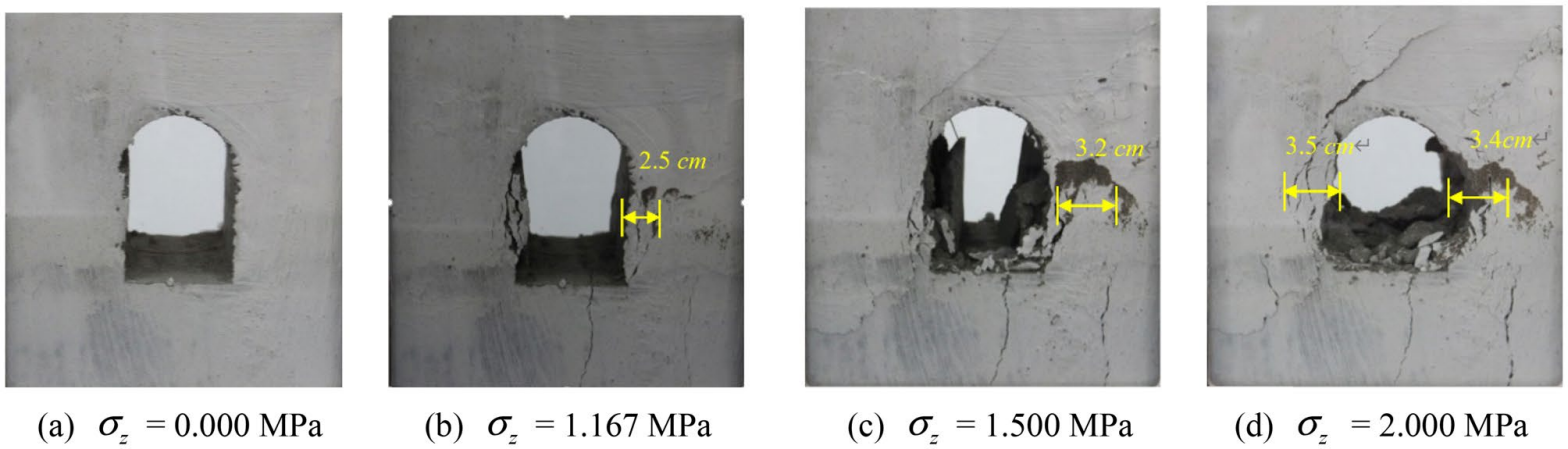
Fracture propagation in the tunnel model during the loading testing.

The fracture propagation in the tunnel model during the loading testing is presented in Fig. 4, with Fig. 3a being the photo of the pre − made tunnel model. The vertical load σ_z_ applied on the top surface of the model was progressively increased after reaching the given strength. No notable change could be detected in the slightly loaded model.

As σ_z_ arose to 1.167 MPa, the initial fractures on the left sidewall grew into open penetrated fractures. Moreover, new crevices appeared in deeper zone with approximate 2.5 cm damage depth. The fracture at the foot of the right sidewall propagated obliquely upward accompanied with several vertical fractures, as shown in Fig. 4b. As σ_z_ growing to 1.50 MPa, the tensile failure zone on the right encountered overall collapse, with a caving depth of 3.2 cm, which is presented in Fig. 4c. It was identified as the primary failure of the model. While the load further increased, the surrounding rock collapsed layer by layer, and the failure planes gradually penetrated towards deeper zone on both sides. The damaged depth of the left side was about 3.4 cm, and that of the right side was 3.5 cm, as shown in Fig. 4d.

**Figure 5:**
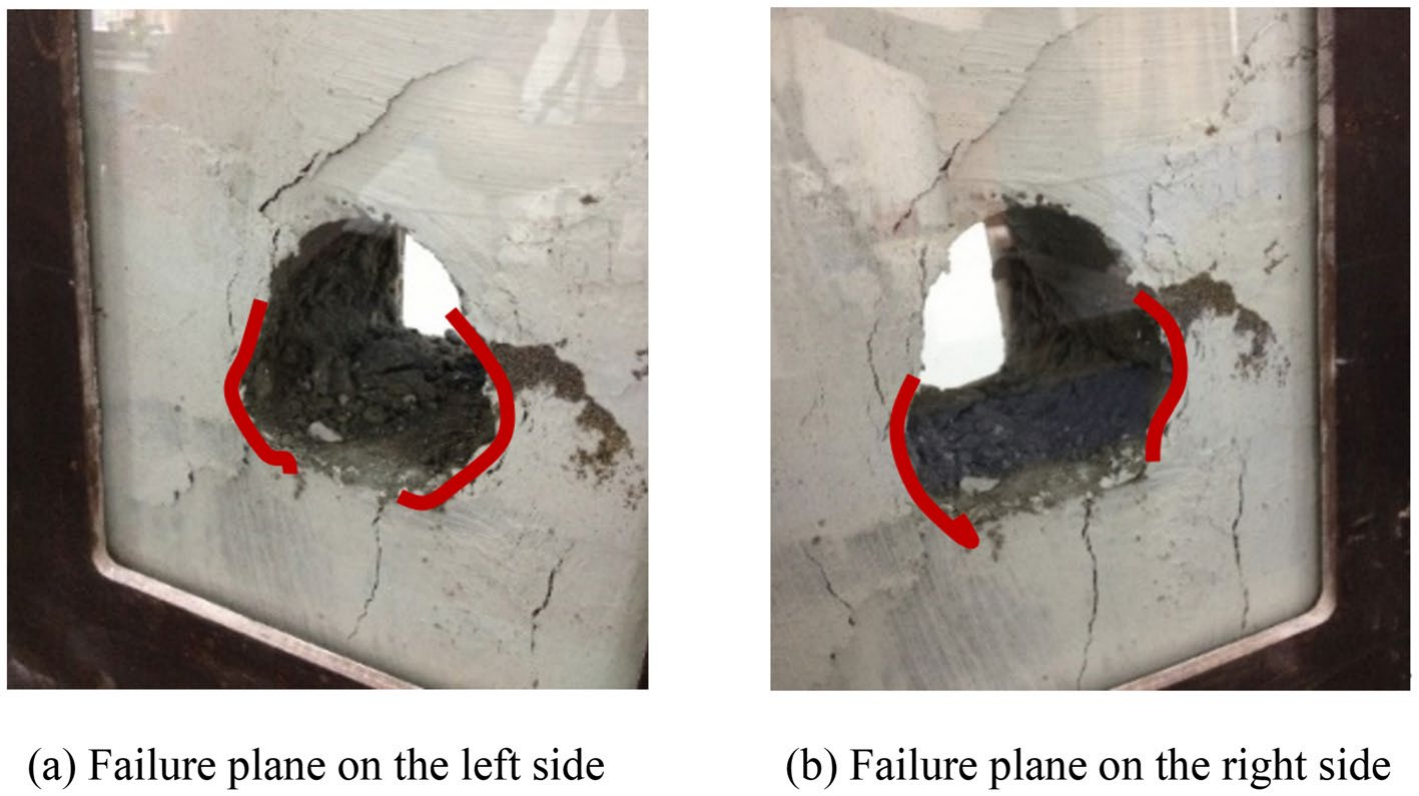
Failure planes of the composite materials tunnel model.

The characteristics of the failure plane are presented in Fig. 5. The model testing indicated that the surrounding rock failure of the tunnel mostly occurred on vertical sidewalls of both sides. With the certain load, vertical fractures initiated at the tunnel vertical walls, then rock collapsed, and the failed area gradually extended towards deeper region of the surrounding rock. Meanwhile, shear fractures propagated-obliquely upward and downward at the feet of both side walls and the spandrel, which cut the tunnel vertical wall into a wedge. Along with the sliding plane of the wedge moved inwards, the fracture aperture grew and several tensile failure planes were generated, which led to layer − by − layer collapse. Then collapse of the V − shaped damaged and extruded sidewall body could be noticed. The failure pattern can be concluded as the splitting off the damaged and extruded sidewall body into a wedge under the joint effect of the shear failure and the vertical tensile failure sidewall, as marked by red lines in Fig. 5.

### Stress − strain variation

**Figure 6:**
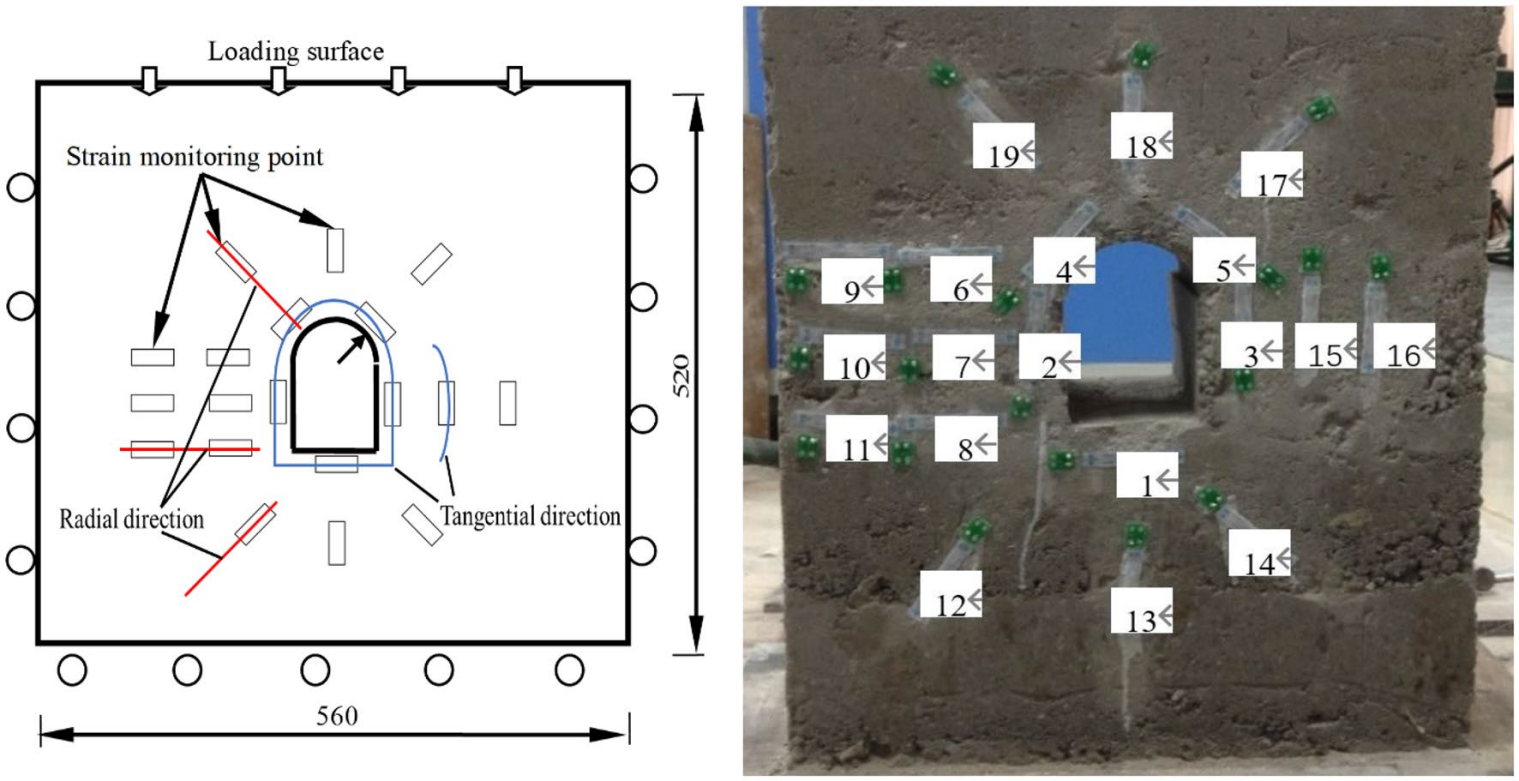
Layout of strain gauges on the testing model.

**Figure 7:**
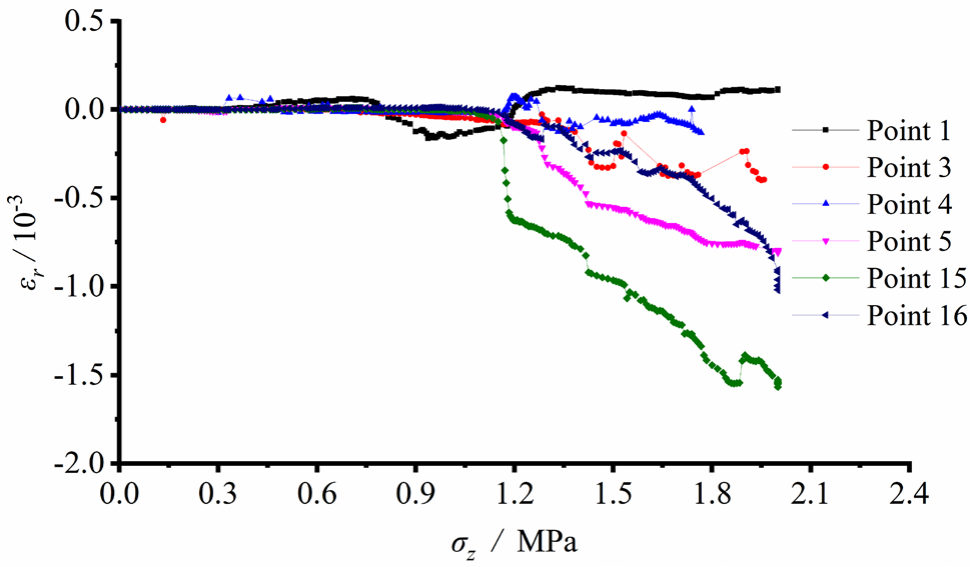
Tangential strain variation vs. vertical load at each measuring spot around the tunnel during loading.

**Figure 8:**
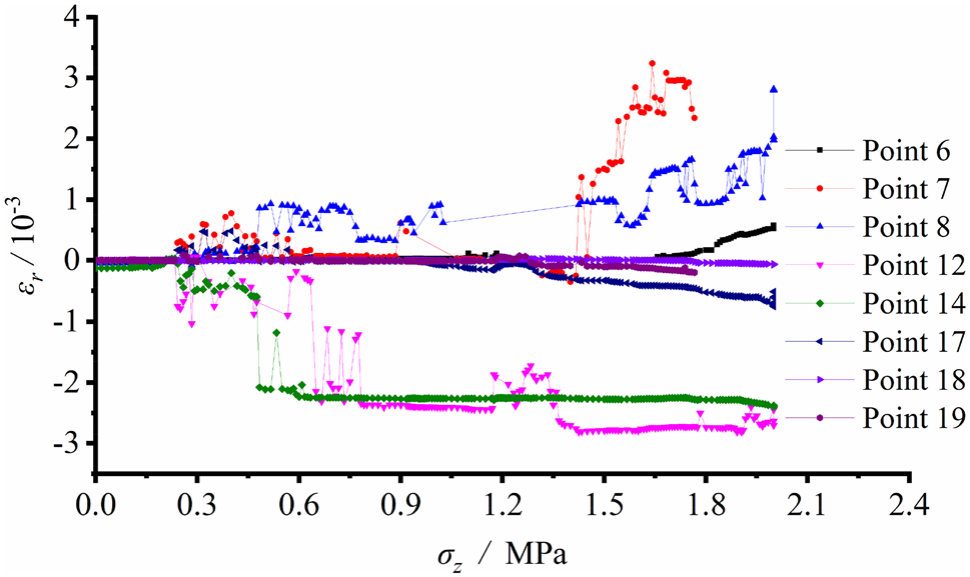
Radial strain variation vs. vertical load at each measuring spot around the tunnel during loading.

During the loading process, the radial and tangential strains at the points distributed on the tunnel wall were recorded. The layout of strain gauges is shown in Fig. 6, the tangential and radial strain variation at each measuring point vs. the vertical load separately plotted in Figs. 7 and 8.

Figure 8 illustrated that Measuring Spot 1 (S1) at the arch bottom during the loading test was in tensile state, yet the absolute value of the tensile or compressive strain at the arch bottom was relatively low (about 1 × 10^−4^). S2, S3, S15 and S16 were in pressured state during loading. The strains slowly increased with the minor load, and S3 had the highest growth rate in strain. As the load grew to 1.167 MPa, the measured strains of S15 and S16 surpassed that of S3 (S15 > S16 > S3), meaning that the strain growth rates of S15 and S16 accelerated and exceeded that of S3. It was the result of fracture development and collapse of the right sidewall, which led to actual load decrease. The tangential measuring spots S4 and S5 z were in pressured states during loading. Their strains slowly grew with relatively minor load, and rapidly increased after the load exceeding 1.250 MPa. At this very moment, fractures initiated at the spandrel.

As presented in Fig. 8, S6, S7 and S8 on the tunnel sidewall under radial strains were in tensile state during loading. With the vertical load reaching 0.333 MPa, the measured tensile strain fluctuating growth was generally figured out and the maximum value happened at S7. As the vertical load reached 1.167 MPa, the tensile strain at each measuring spot showed upheavals followed with irregular fluctuation, which indicated that the strain gauges on the sidewall have broken. This phenomenon was consistent with the vertical tensile fracture on the vertical wall, as shown in Fig. 4b. The radial strains of S17, S18 and S19 at the arch crown were under the pressured state during loading. The compressive strain slowly grew without notable failure upheaval, suggesting the stability of the arch crown. The radial strains of S12 and S14 at the arch bottom were also in pressured states during loading. Fractures initiated at the arch bottom, as the vertical load grew to 0.5 MPa. After a considerable growth in the compressive strain, it still remained stable indicating the expansion of failed zone at the arch bottom stopped.

It was revealed from the strain record that the arch bottom along its tangential direction and sidewall along its radial direction were in tensile state, which led to initiation and propagation of fractures. The failure pattern transformed from the tensile fracture of the arch bottom with minor load into collapse of V − shaped damaged and extruded sidewall led by joint tensile and compressive effects. Figure 8 also identifies the peak load with reference to the loading failure was 1.50 MPa.

### Unloading failure testing

#### Unloading failure testing with surrounding rock stress of 60% σ_zmax_

**Figure 9:**
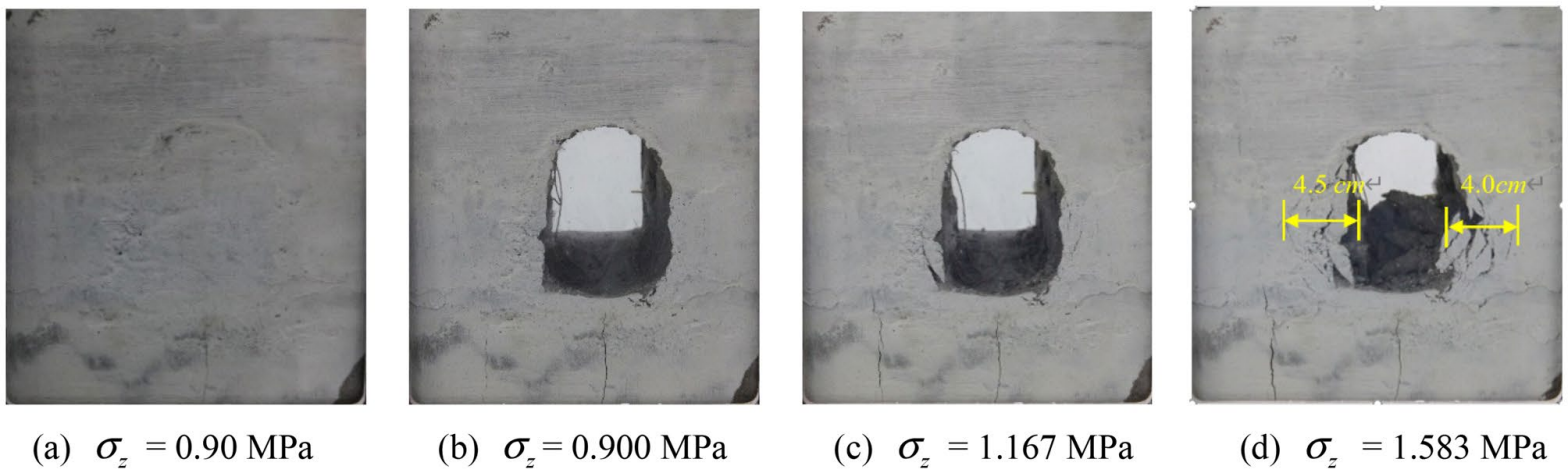
Fracture propagation during excavation unloading with surrounding rock stress of 60% σ_zmax_.

The fracture propagation in tunnel model during unloading is shown in Fig. 9. Exerted 60% previously measured peak load, namely 0.90 MPa, on the model and maintained constant. The excavation started from one side of the model towards the opposite side. Several fractures were found at the foot of the left sidewall and a length of 1.5 cm vertical fracture occurred at the 2.5 cm depth inside the right sidewall. As the width of the fracture at the arch bottom enlarging, a certain excavation − induced damaged zone was created. However, the tunnel surrounding rock as a whole remained steady.

The vertical load was then continuously developed in order to further observe the tunnel failure propagation. As the vertical load σ_z_ reached 1.167 MPa, several short vertical fractures were discovered in the middle of the left sidewall, and the fractures at the foot of the sidewall propagated vertically. Fractures penetrating through the model were came into being at the right spandrel, and small particles continuously dropped from the foot of the sidewall and the spandrel, as shown in Fig. 9c. With load of 1.583 MPa, V − shaped wedge shear planes appeared on two sides of the tunnel. The collapse depth of the left vertical wall was 4.5 cm, and that of the right was 4.0 cm, as shown in Fig. 9d. The wall successively collapsed, and the failure zone gradually extended toward the deeper zones on both sides.

Only a small fractured zone was found around the sidewall during unloading with surrounding rock stress of 60% σ_zmax_. After the accomplishment of excavation, the exerted load started to grow. Vertical split fractures were generated in the middle of the sidewall meanwhile oblique shear fractures initiated from the foot of the wall and the spandrel, which cut the tunnel vertical wall into a wedge. The wedge sliding plane moved inwards, the width of the vertical tensile fracture on the sidewall grew, and consequently the wall collapsed layer by layer. The failure pattern presented itself as collapse of the V − shaped damaged and extruded sidewall under the joint effects of the shear failure and the vertical tensile failure of the sidewall wedge.

**Figure 10:**
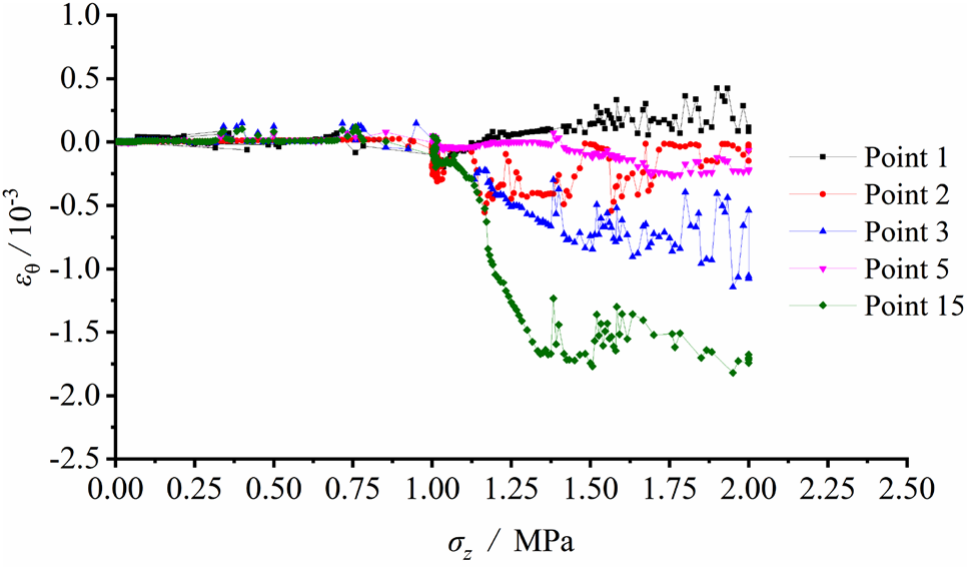
Tangential strain variation vs. vertical load at each measuring spot around the tunnel during unloading with surrounding rock stress of 60% σ_zmax_.

**Figure 11:**
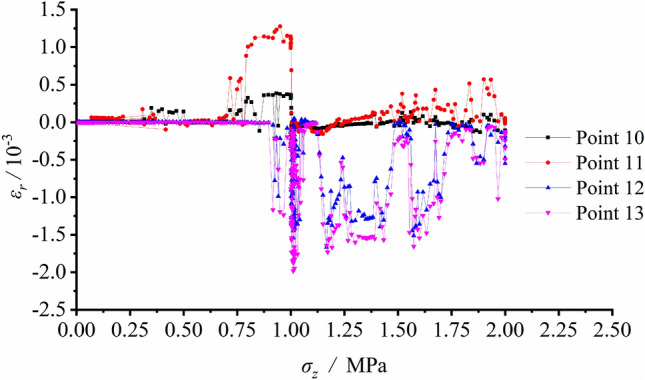
Radial strain variation vs. vertical load at each measuring spot around the tunnel during unloading with surrounding rock stress of 60% σ_zmax_.

The radial or tangential strain variation at each measuring spot were recorded during the excavation unloading process. The strain gauges layout is shown in Fig. 6, the tangential strain and radial strain versus vertical load are illustrated separately in Figs. 10 and 11.

Figure 10 indicates small deformation and corresponding strains in low values at each spot and shows only limited increments of strains after excavation. Afterwards, the loading process began. The tangential strain at S1 was in tensile state. S2, S3 and S5 on the sidewall were in pressured state during loading, which implied that the tunnel wall was extruded towards the free face. The comparison of the measured strains between S3 and S15 showed the strain values were basically equal prior to the load of 1.167 MPa. However, as the load further grew, the strain growth rate at S15 considerably exceeded that at S3. This meant that the stress magnitude and the strain growth rate declined at last, as the position approaching the failed loosened zone.

According to Fig. 11, tensile stress was generated by excavation disturbance at S10 and S11 in the deep regions of the sidewall. The tensile strains reached their maximum values after excavation, suggesting that the two vertical sidewalls were in tensile state. Relatively minor strains were observed at arch bottom measuring points S12 and S13, and it was safe to say that the arch bottom was stable.

The measured strain variation illustrated that the deformation and strain were relatively small before tunnel excavation. As excavating, the strains at each measuring spot greatly grew. The arch bottom was subjected to tangential tension and the vertical sidewall along the radial direction was also in tensile state, which caused initiation and growth of the fracture. Meanwhile, shear fractures initiated at the foot of the wall and the spandrel, this resulted in the great compressive deformation of surrounding rock and extruding towards the free face. The sidewall suffered from the V − shaped collapse of its damaged and extruded part under the combined shear and tensile effects.

### Unloading failure testing with surrounding rock stress of 100% σ_zmax_

**Figure 12:**
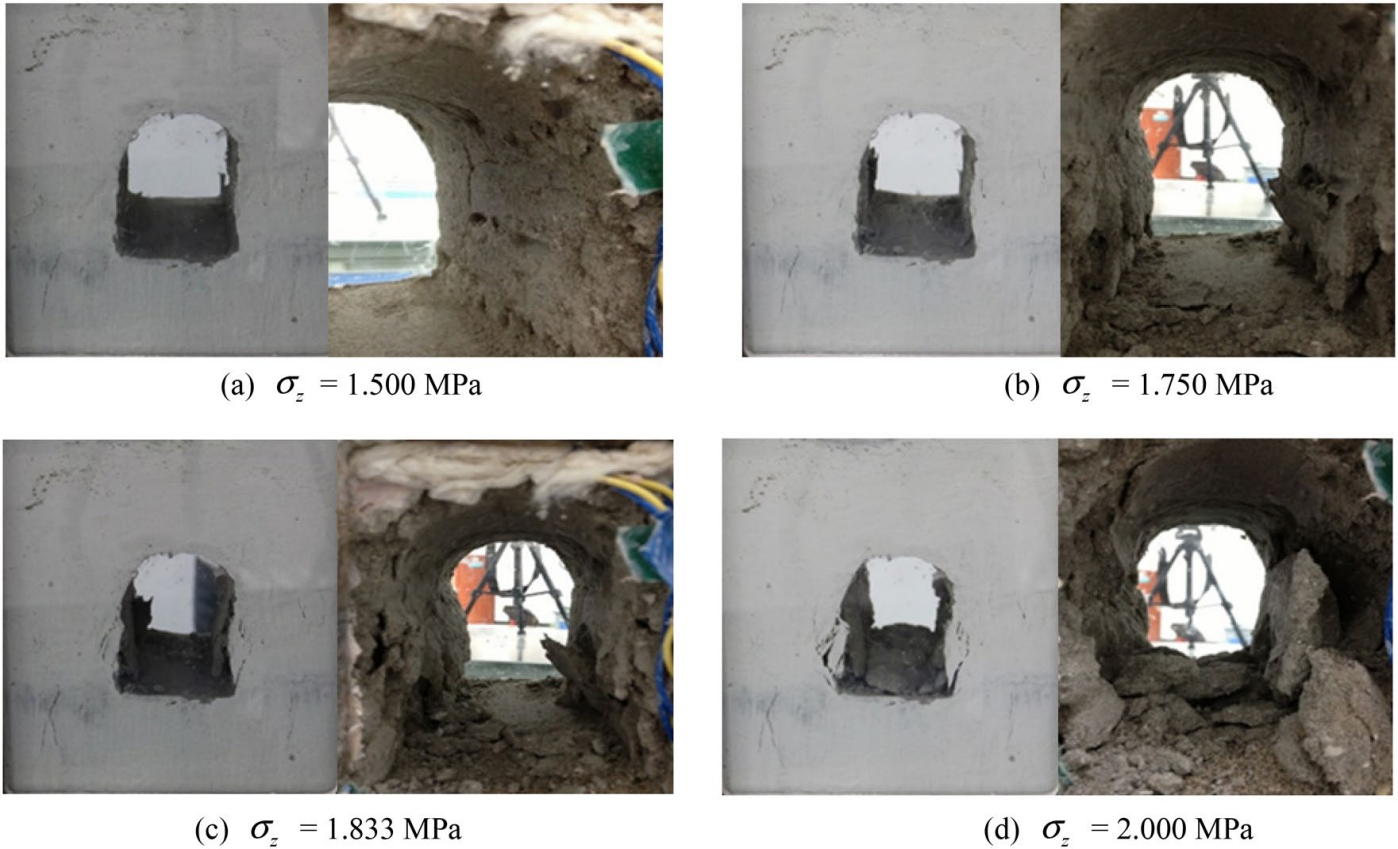
Fracture propagation during excavation unloading with surrounding rock stresses of 100% σ_zmax_.

The fracture propagation inside the tunnel model during unloading with surrounding rock stress of σ_zmax_ is shown in Fig. 12. The initial surrounding rock stress σ_z_ of 1.500 MPa was firstly imposed on the model before excavation. Then, with constant surrounding rock stress, the excavation proceeded from one side of the model and penetrated towards the other side. No notable fractures appeared in the surrounding rock, and the whole structure was basically stable, as presented in Fig. 12a.

A loading process was followed in order to further reveal the failure propagation in the surrounding rock. Shear fractures developed obliquely upward from the feet of the two sidewalls, and further extended upward. The depth of left vertical fractures were about 1.0 cm, and the surrounding rock of the vertical sidewall extruded towards the free face and ultimately collapsed (Fig. 12b). As load reached 1.833 MPa, the surrounding rocks of the two vertical sidewalls collapsed due to forced extrusion towards the free face, and shear failure planes penetrated through the V − shaped wedge from the foot of the wall to the spandrel, as shown in Fig. 12c. Collapse successively occurred, as the load proceeding, and the failure plane further developed into the deeper region of the model. Figure 12d presents that the third collapse layer led to fractures penetrating through the whole model.

The excavation − induced unloading caused the damage and fracture of surrounding rock and rock failure mostly occurred on the sidewall of the tunnel. The loading process following excavation generated vertical split fractures near the sidewall. Meanwhile, oblique shear fractures were formed at the foot of the wall and the spandrel, which cut the vertical wall into a wedge. The wedge sliding plane moved inwards and the vertical fracture width on both sides of the vertical sidewalls grew. Correspondingly, several tensile fractures came into being and resulted in the layer − by − layer collapse of the wall. The failure pattern could be summarized as collapse failure of the V − shaped extrusion of the damaged wall under the joint effects of the shear failure and the vertical tensile failure of the sidewall wedge.

**Figure 13:**
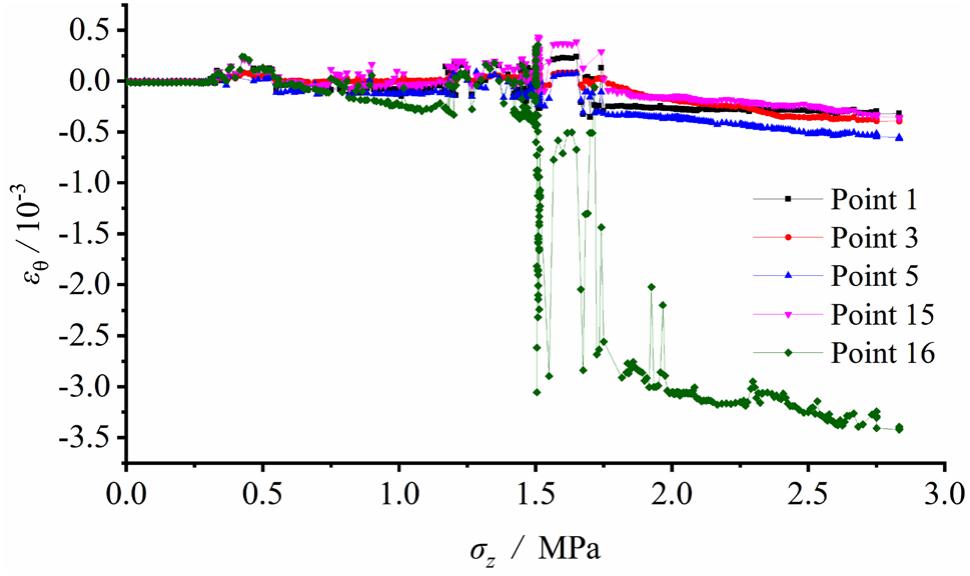
Tangential strain variation vs. vertical load at each measuring spot around the tunnel during unloading with surrounding rock stress of 100% σ_zmax_.

**Figure 14:**
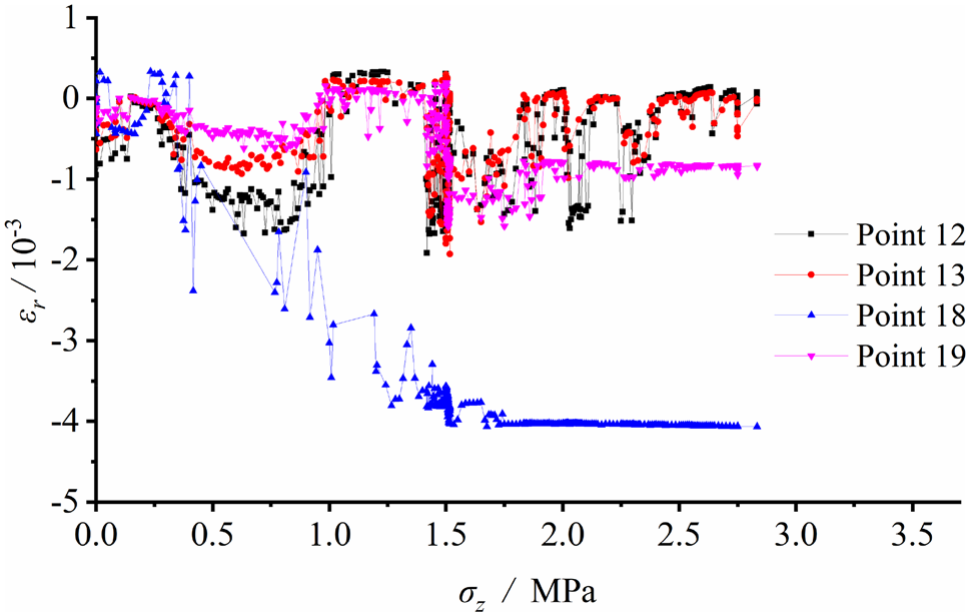
Radial strain variation vs. vertical load at each measuring spot around the tunnel during unloading with surrounding rock stress of 100% σ_zmax_.

The radial and tangential strains at each measuring spot around the tunnel were logged during unloading. The tangential strain variation versus the load at each spot is plotted in Fig. 13, while that of the radial strain is shown in Fig. 14.

Figure 13 shows the relatively small strain at each measuring spot before excavation, indicating the deformation of the model was in a small value. As excavation proceeding, a certain and limited strain fluctuation could be seen at each spot owing to the true embodiment of excavation disturbance. During the last loading process, the measuring spots around the tunnel were in pressured state, suggesting the tunnel was extruded towards the free face after excavation. The radial strains at S12, S13, S18 and S19 are illustrated in Fig. 14. Due to excessive stress, the tensile strains at S6 − S11 of the sidewalls set for radial strain measurement exceeded the range of the strain gauge, which meant the failure depth of the surrounding rock of the two vertical sidewalls increased with the vertical load. S12 and S13 at the arch bottom were in pressured state, yet with minor strain values. A certain reductant amount of compressive strain could be observed after the overall collapse of the entire structure. The strain at the arch crown generally stayed stable during excavation and subsequent loading process.

The strain variation also suggested that prior to excavation, the model deformation was negligible. The strain at each spot around the tunnel grew after excavation. The arch bottom along the tangential direction and the sidewall along the radial direction were in tensile state, which contributed to the occurrence and propagation of vertical fractures. Meanwhile, shear fractures were generated at the foot of the sidewall as well as the spandrel, and the surrounding rock was extruded towards the free face, which ultimately evolved into a failed loosened zone. The stress magnitude inside the loosened zone declined.

### Comparison between loading and unloading

**Figure 15:**
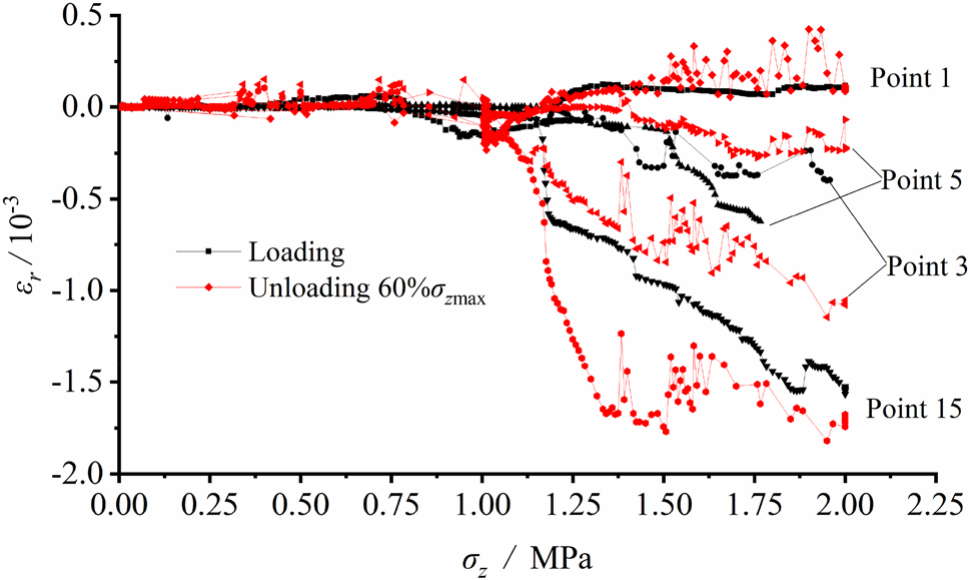
Tangential strain variation at each measuring spot around the tunnel in loading and unloading testing.

The tangential strain variations at each measuring spot around the tunnel in loading and unloading testing are shown in Fig. 15, with the black line representing the case of loading testing and the red one standing for the unloading testing with surrounding rock stress of 60% σ_zmax_. It illustrated that during the loading failure testing, the strain was relatively small when the value of load was low. As the load grew to 0.833 MPa, the strain increased, and its growth rate accelerated after the load reaching 1.250 MPa. As for the excavation unloading process, the strain of the surrounding rock was relatively small prior to excavation and it rapidly grew during excavation with the imposed constant load of 0.900 MPa.

The comparison between the loading and unloading revealed that S1 at the arch bottom presented a certain amount of tensile strain in both cases and the strain values were both basically constant in small values. However, the strain growth rates at S3, S5 and S15 during unloading exceeded those during the loading process. It implied that the deformation rate of the surrounding rock extruding towards the free face during unloading was faster than that in the loading case and consequently the failure evolution accelerated.

Comparison between model testing and DEM simulation.

**Figure 16:**
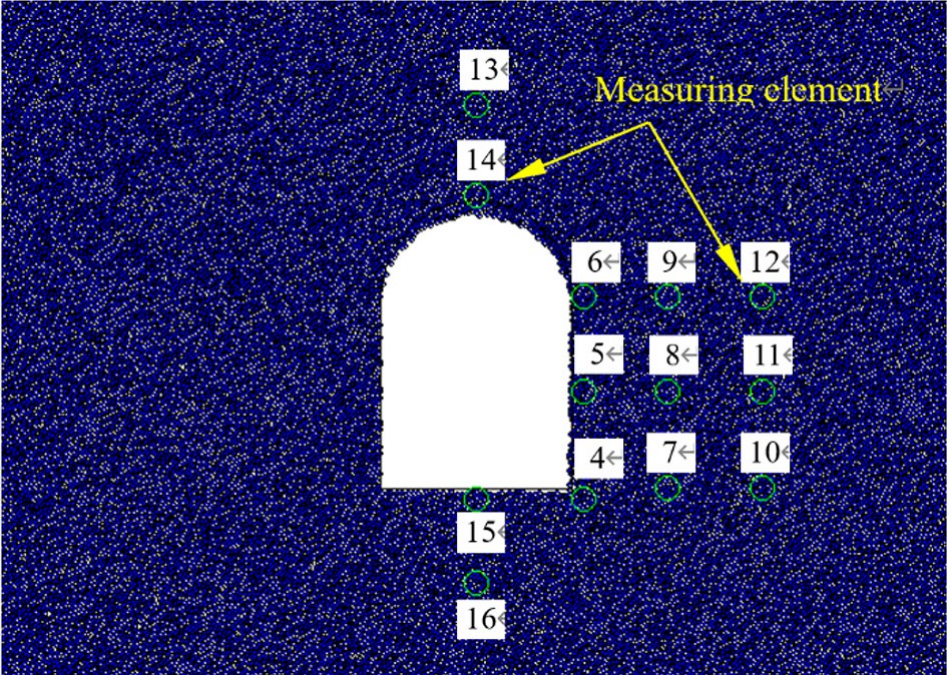
Simulation model.

A particle flow model that is identical to the physical model is built using the FISH programming in PFC^2^^D^ so as to compare the results of the physical tunnel model and PFC numerical testing. The numerical model is shown in Fig. 16. The simulation model was simplified into a plane − strain model with constraints imposed against the left, right and bottom surfaces of the model. A servo − controlled loading on the top surface was implemented to facilitate the loading failure of the tunnel and thus mimicked the actual testing process. The accuracy of the discrete element method (DEM) − based simulation is directly influenced by the given value of the microscopic parameter. This paper compares the mechanical parameters obtained from the laboratory and numerical triaxial testing, and thoroughly analyzes the effects of each microscopic parameter of the parallel bonded model on the macroscopic mechanics by changing only one factor at a time. The regularity behind the interaction between each parameter was revealed, and the final values of the microscopic mechanic parameters are presented in Table 5. The microscopic constitutive model adopted the contact − stiffness model.

**Table 5:** PFC Microscopic parameters.

Porosity *n*/%	Minimum particle size *R*_*min*_/mm	Radius ratio *R*_*max/*_*R*_*min*_	Particle density *ρ*_*b*_(kg/m^3^)	Particle contact modulus *E*_*c*_ /GPa	Particle stiffness ratio *k*_*n*_*/k*_*s*_*(1)*	Friction coefficient *u/1*	Bonding radius multiplier $\overline{\lambda }$/1	Parallel bond modulus $\overline{E}$_*c*_/GPa	Parallel bond stiffness ratio $\overline{k}$_*n*_/$\overline{k}$_*s*_*(1)*	Normal bonding strength $\overline{\sigma }$_*c*_/$\overline{\sigma }$_*cs*_(MPa)	Tangential bonding strength $\overline{\tau }$_c_/$\overline{\tau }$_cs_(MPa)
16	0.30	1.66	2 143	40.5	2.00	0.60	1.0	24.0	2.00	1.15/0.01	0.82/0.01

### Damage propagation during loading testing

**Figure 17:**
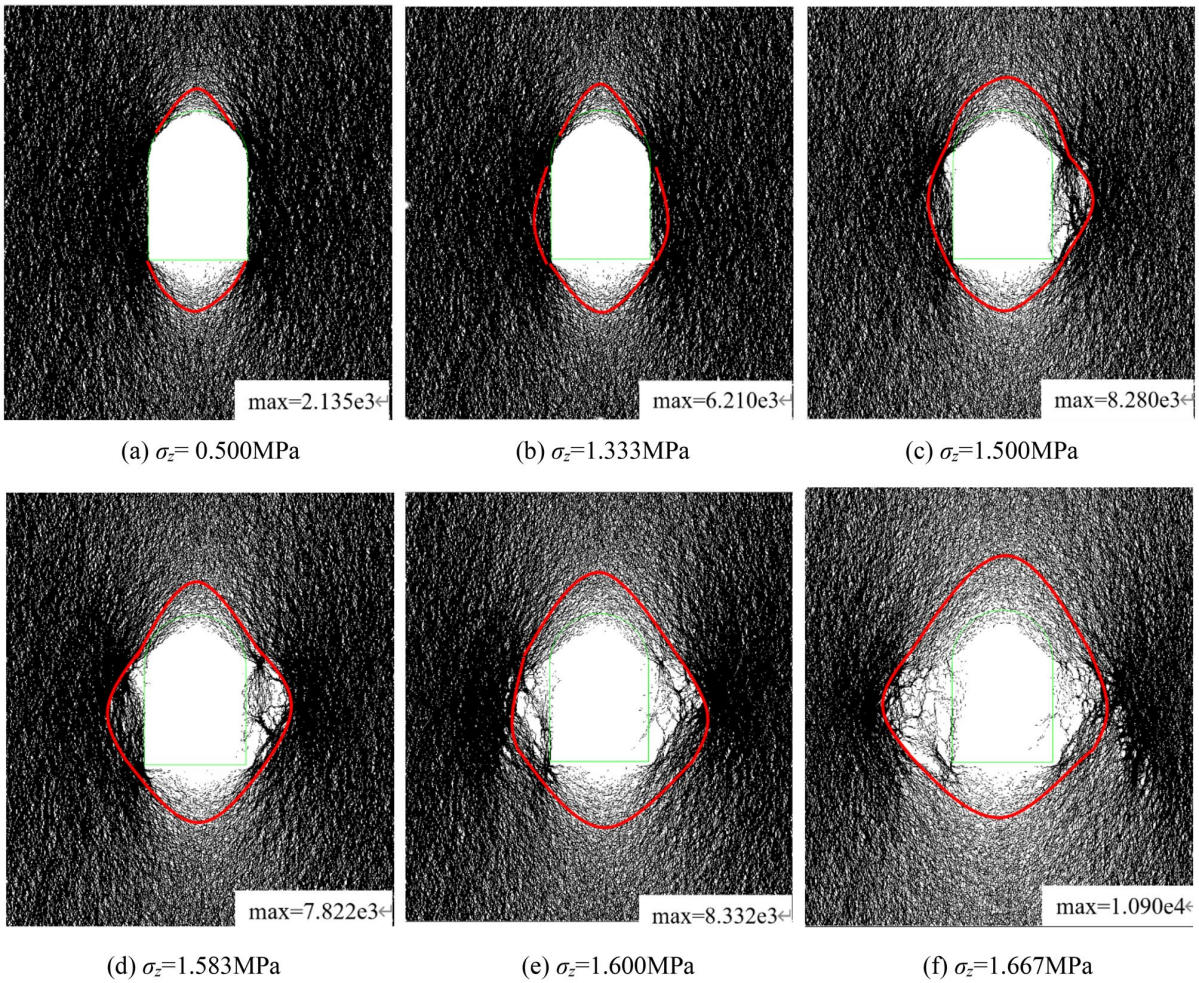
Force chain distribution during the loading failure testing.

The force chain distribution of the tunnel during the loading testing is illustrated in Fig. 17. It showed that during the early loading stage, strong force chains were distributed along the free face of the vertical wall and the arch crown and bottom were in pressured state, as marked by red lines in Fig. 17a. As the imposed vertical load rose from 0.5 MPa to 1.167 MPa, the contact load between particles gradually grew without notable variation with respect to the force chain distribution: the strong force chain still presented itself on and paralleled to the free face of vertical wall. With vertical load reaching 1.333 MPa, the force chain around both sidewalls was bent and the strong force chain moved towards the deeper surrounding wall, as presented by red lines in Fig. 17b. This suggested that a certain unloading loosened zone was created at both sidewalls, moreover the depths of the pressure arches at arch crown and bottom basically remained constant. The loosened zone at the two vertical sidewalls as well as the pressure arch depth of the arch crown further enlarged with the increasing load. However, the growth rate of the pressure arch depth was inferior to the expansion rate of the loosened zone of the vertical wall. In addition, the range of the pressure arch at the arch bottom slightly changed. As the load continued growing to 1.500 MPa, the pressure arch depths at the arch crown and bottom both climbed up, meanwhile the contact stress in the surrounding rock of the two vertical sidewalls considerably declined, indicating that a loosened zone formed in this area. sidewall and the whole surrounding rock on both sides of the tunnel was loosened as the vertical loading reaching 1.600 MPa. The strong force change migrated towards the deep position of surrounding rock and the loosened zone further expanded.

**Figure 18:**
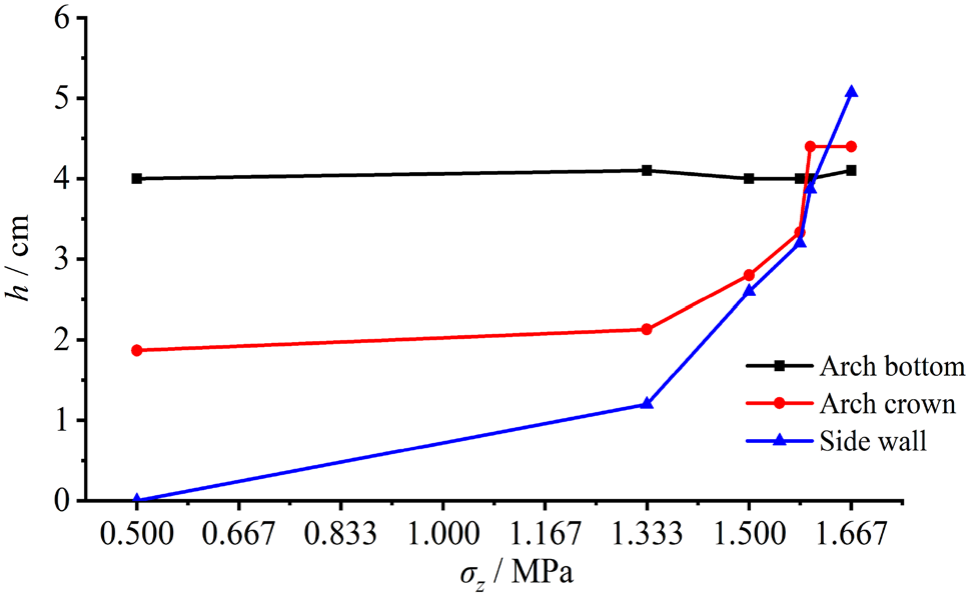
The range variation of the loosened zone (pressure arch) with the increasing load.

Figure 18 presents the range variations of the pressure arches at the arch crown and bottom as well as the loosened zones around the two vertical sidewalls. As it is illustrated, the depth of the pressure arch at the arch bottom basically remained constant. In terms of the vertical load was lower than 1.333 MPa, the pressure arch at the arch crown and the loosened zones around the two vertical sidewalls slowly enlarged yet such expansion dramatically accelerated as load continued increasing. As the load reached 1.600 MPa, a notable growth of the loosened zone depth suddenly appeared, indicating that the tunnel failure (peak) load was 1.600 MPa.

**Figure 19:**
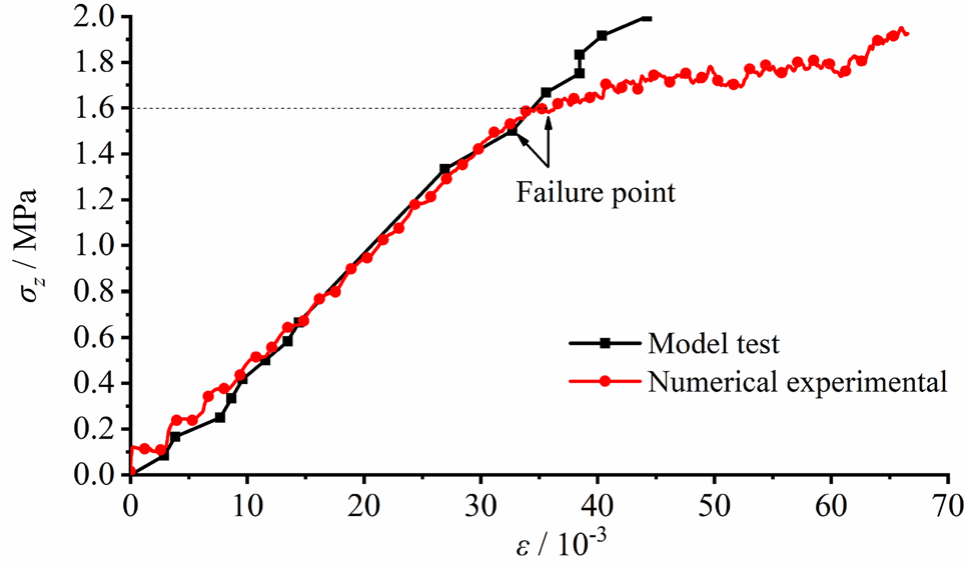
Comparison of load − deformation correlations between laboratory and numerical loading testing.

The comparison of load − deformation correlations is shown in Fig. 19, in which black line indicates the laboratory testing results and red line represents numerical testing results. It was revealed that during the initial stress applying stage, an elastic correlation was found on the top surface between deformation and load and the data curves of laboratory and numerical testing basically coincided with each other. The peak load observed in the laboratory testing was 1.500 MPa with a corresponding strain of 33 × 10^−3^, while the peak load of the numerical testing was 1.600 MPa with a corresponding strain of 36 × 10^−3^. The loads and strains for the primary failure were quite closed. After the primary failure, the load vs. strain curve of the laboratory testing bent, but the model could still sustain higher load. In the case of the numerical testing, the model strength dramatically declined after primary failure. This contradiction was mainly attributed to the fact that the strength of the physical model material was improved by model compaction induced by load, while the material strength of the numerical testing was invariable. The stress vs. strain curve of the numerical testing gradually deflected from the linear elasticity. And as it reached the peak, it finally fell in a curved manner. The elastic modulus and the peak load observed in the laboratory testing were higher than those in the numerical testing.

**Figure 20:**
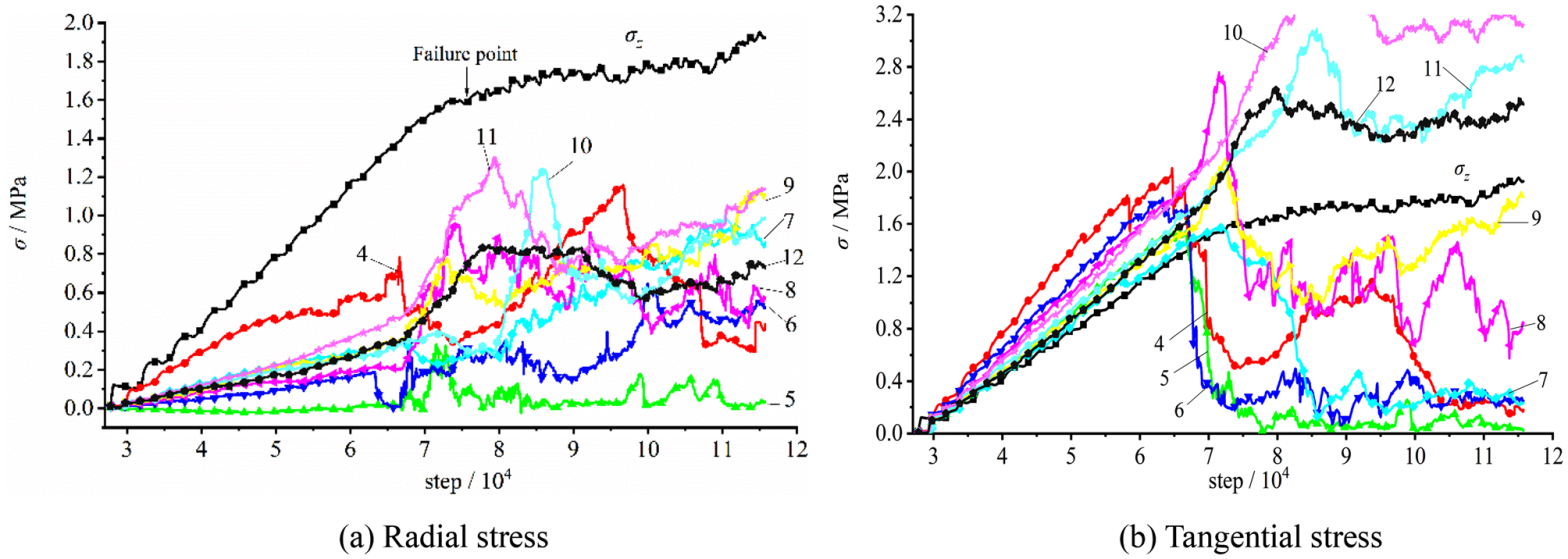
Stress variation of the measuring element on the vertical sidewall during the loading testing.

The stress variation captured by measuring elements on the vertical sidewall during the loading testing is plotted in Fig. 20. It can be figured out that during the gradual loading of the initial stress, the stress at each measuring element grew linearly. The tangential stress σ_θ_ was about 1.05 − 1.80 times larger than the imposed surface stress σ_z_, meanwhile the radial stress σ_r_ is about 0.12–0.62 times smaller than the surface stress σ_z_, which suggested that the radial and tangential stresses near the free face during the loading testing increased with varied growth rates. When the top surface stress reached 1.333 MPa, the stress of Measuring Element 4 (E4) peaked (σ_r_ = 0.78 MPa, and σ_θ_ = 2.03 MPa) and dropped afterwards, suggesting that damage occurred at the interior of the model. The stresses at E5 and E6 reached their peaks, with σ_z_ = 1.367 MPa, σ_θ_ = 0.83 MPa and 1.28 MPa, respectively. Also, the stresses quickly declined to low values. This reflected that the surrounding rock of the free face was failed and loosened. The maximum tangential stress at E7 appeared with the imposed load of 1.533 MPa and σ_θ_ = 1.57 MPa and fell afterwards. The stress at E9 got the maximum value at σ_z_ = 1.55 MPa and σ_θ_ = 2.10 MPa. The maximum tangential stress at E7 occurred with σ_z_ = 1.590 MPa and σ_θ_ = 2.14 MPa and declined as the loading proceeded. This illustrated that the loosened zone of the surrounding rock constantly expanded. As iteration proceeding, the measuring spot located in deeper surrounding successively encountered its peak stress and declined subsequently, with the failed and loosened zone of the surrounding rock continuously expanding.

**Figure 21:**
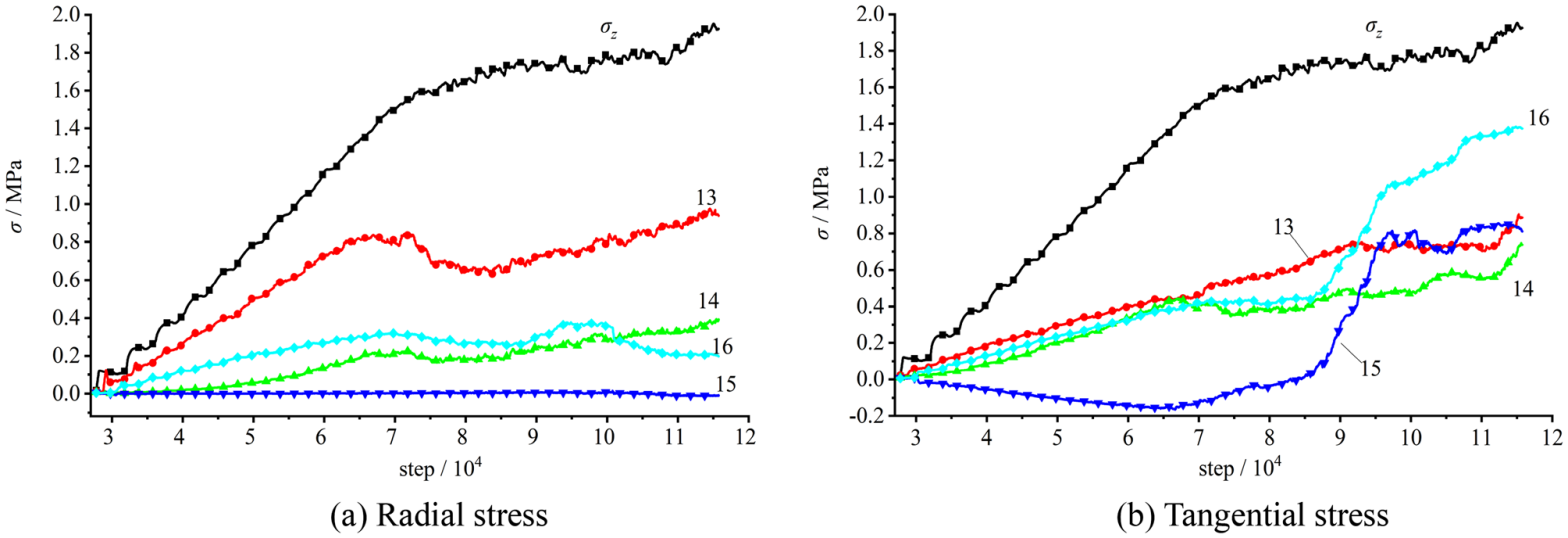
Stress variation of the measuring element at the arch crown and bottom during the loading testing.

The stress variation of the measuring element at the arch crown and bottom during the loading testing is presented in Fig. 21, which illustrated the radial and tangential stresses of each measuring element were always smaller than the stress imposed on the top surface of the model σ_z_.

In terms of the radial stress, the stress value at each measuring element was different with each other. The stresses at E13 and E16 in the interior of the surrounding rock exceeded those of E14 and E16 on the tunnel surface, which meant the radial stress gradually rose from the tunnel surface to the deeper surrounding. The radial stress of E15 at the arch bottom was basically fixed at zero and those of E13 − E16 reached their peak then remained constant afterwards as the imposed stress grew to 1.392 MPa. As for the tangential stress, E15 was in tensile state while the model was loaded then peaked at 0.163 MPa under the vertical load of 1.386 MPa. The tangential tensile stress then declined and even transformed into the pressured state upon the arrival of the peak load. This demonstrated that a certain amount of tensile stress occurred at the model bottom owing to the loading. However, the tensile stress basically stayed stable during the iteration, which complied with the phenomenon in the laboratory model testing. Moreover, compressive stresses of E13, E14 and E16 were almost equal to 30% σ_z_ and the stresses grew synchronously with the increasing load on the top surface which indicated that the stress states of the arch crown and bottom were basically stable.

With continuous loading after the primary failure of the surrounding rock, obvious inflection points could be figured out in the stresses of E15 and E16 at the arch bottom, as σ_z_ surpassed 1.72 MPa (the peak load). The stresses of former two elements grew rapidly, while the stresses of E13 and E14 at the arch crown maintained its previous growth rate. Consequently, this gave rise to the overall instability and failure of the tunnel model.

### Damage propagation during the unloading testing

**Figure 22:**
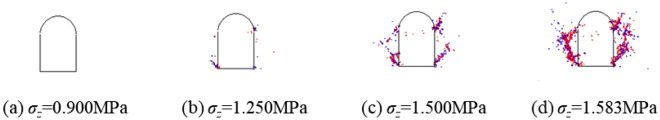
Damage propagation during excavation unloading with imposed vertical stress of 0.900 MPa.

The damage zone propagation, corresponding to the excavation unloading testing with a constant vertical stress of 60% σ_zmax_ firstly imposed on the top surface of the model, is presented in Fig. 22. There was It was intact prior to tunnel excavation. As the excavation initiated, the model showed no apparent failure during the iteration. However, as the imposed load further grew to 1.250 MPa after excavation, the damaged zones occurred at the feet of the sidewalls and the spandrel. With load of 1.500 MPa, fractures originated from the foot of the sidewall and propagated obliquely upward towards the deeper surrounding rock, while the fracture at the spandrel extended obliquely downward. Moreover, a small quantity of particle mass dropped from the free face. As the load reached 1.583 MPa, the fractures at the foot of the sidewall and the spandrel joined together and created penetrated failure. The fracture width of the failure plane enlarged after the continuing iteration. The failed zones at the two sidewalls expanded deeper into the surrounding rock, which conducted new sliding planes.

**Figure 23:**
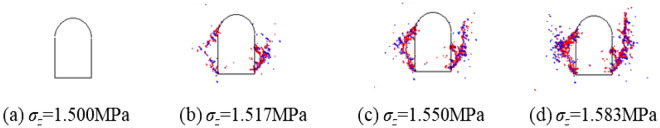
Damage propagation during excavation unloading with imposed vertical stress of 1.500 MPa.

Figure 23 illustrates the damage propagation during the excavation unloading process with the constant vertical stress of 100% σ_zmax_ firstly imposed on the model prior to excavation. Before excavation, the tunnel model was intact. The damage zones were found at the foot of the sidewall and the spandrel respectively, as the excavation initiated. During the loading process after excavation, the damage zones at the foot of the wall and the spandrel expanded, with fractures at the foot of wall propagating obliquely upward into the deeper surrounding rock and fractures at the spandrel extending obliquely downward. The fractures at the foot of the wall and the spandrel joined together and formed a penetrated failure plane, as the imposed load reached 1.550 MPa. The fracture width of the sliding plane expanded with the increasing load, and the failed zones at the two sidewalls expanded towards the interior of the surrounding rock, which generated new sliding planes.

**Figure 24:**
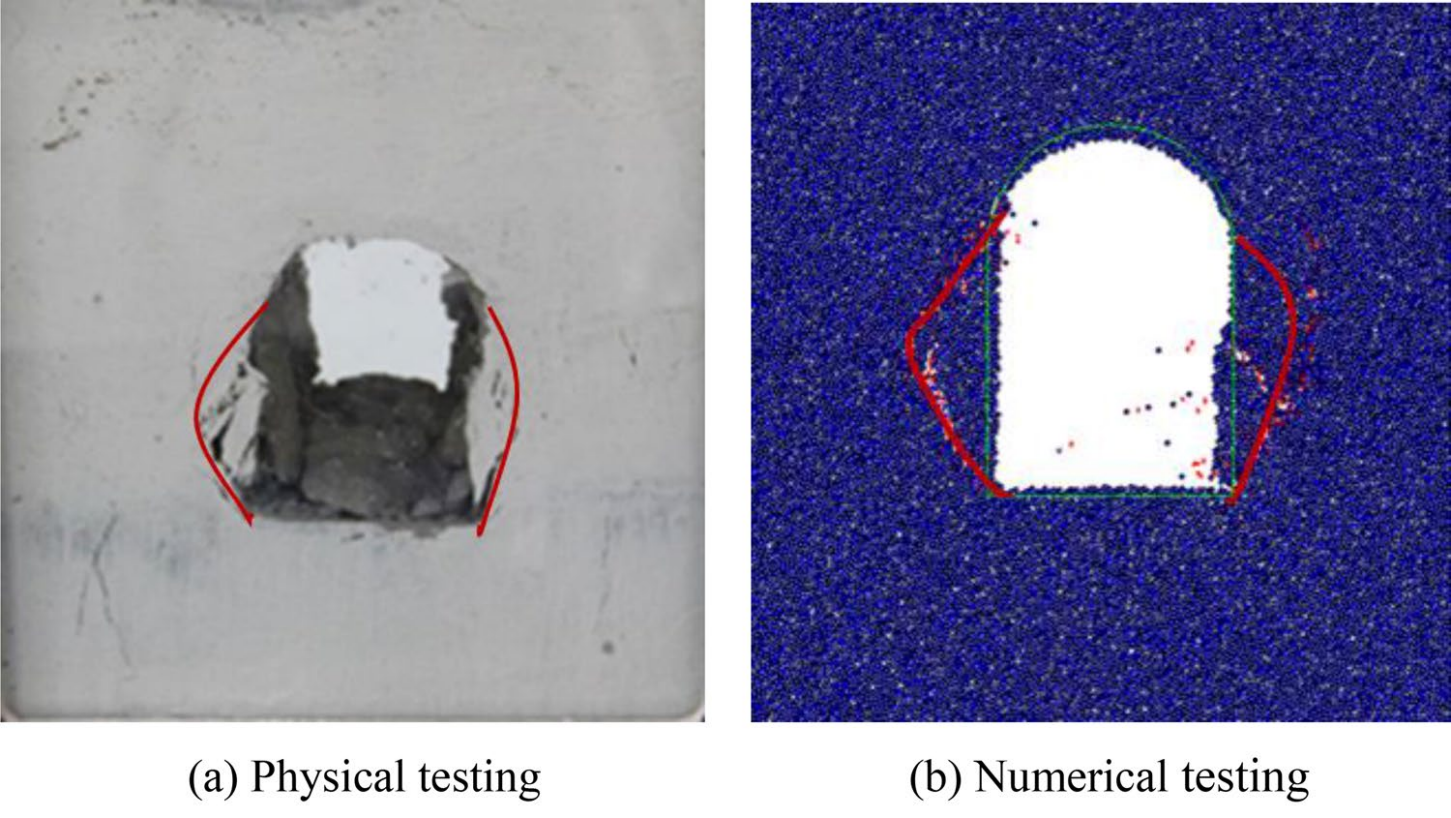
Comparison of failure planes of the physical and numerical excavation unloading testing.

The failure plane obtained in the laboratory was compared with that of numerical excavation unloading testing, as shown in Fig. 24. V − shaped wedge shear planes were presented on the two sidewalls in the laboratory testing, while penetrated failure planes from the foot of the sidewall to the spandrel area were found in the numerical testing. In the physical model testing, the collapsed rock mass broke in the middle due to deterred sliding of the lower part rock mass, and its integrity of the dropped rock mass was poor, while the sliding body in the numerical testing maintained relatively high integrity. This was because the material used in the particle flow modeling was homogenous. Moreover, its tensile strength was higher than that of the physical model, which conducted inadequate development of tensile fractures. However, it was worth pointing out that the failure patterns, the regularity of failure plane propagation and failure plane ranges in both cases were in good agreement.

**Figure 25:**
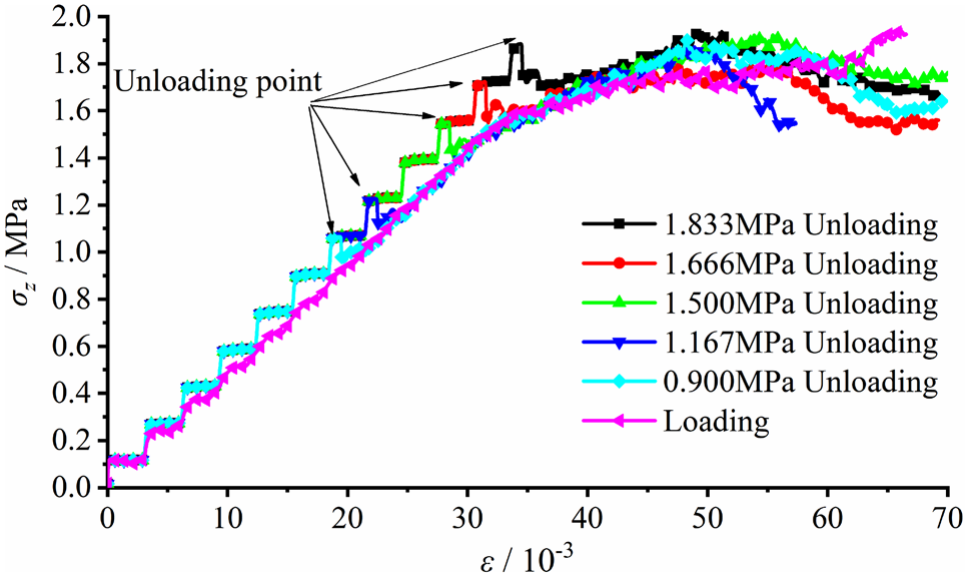
Load vs. deformation in the model with varied initial surrounding rock stresses.

The load vs. deformation curves with varied initial vertical surrounding rock stresses are plotted in Fig. 25. During the exertion of the initial rock stress before excavation, the load on the top surface presented wavelike rise, and had basically linear (elastic) correlation with strain. Compared with the loading testing, the elastic modulus in the linear elastic section as well as the amplitude of the fluctuation of the unloading testing curve was slightly larger. As the stress reached the set value, excavation began and the stress vs. strain curve had a small drop in both cases. As far as the initial surrounding rock stress was lower than the peak load σ_zmax_, the tunnel didn’t encounter failure during excavation, and the stress vs. strain curve during the subsequent loading process almost coincided with that of the loading testing. After the initial rock stress exceeding the peak load, the stress in the model didn’t grow during excavation but the strain rose up, which led to the fast observation of the primary failure penetrated through the tunnel. The stress vs strain curve continued climbing up with a lower slope instead, during the subsequent loading process after the primary failure. This could be mainly attributed to the reduction of the elastic modulus of the surrounding rock at the two sidewalls induced by the damage and collapse. In the meantime, new profiles of the tunnel were formed, which required higher load to facilitate next tunnel failure.

**Figure 26:**
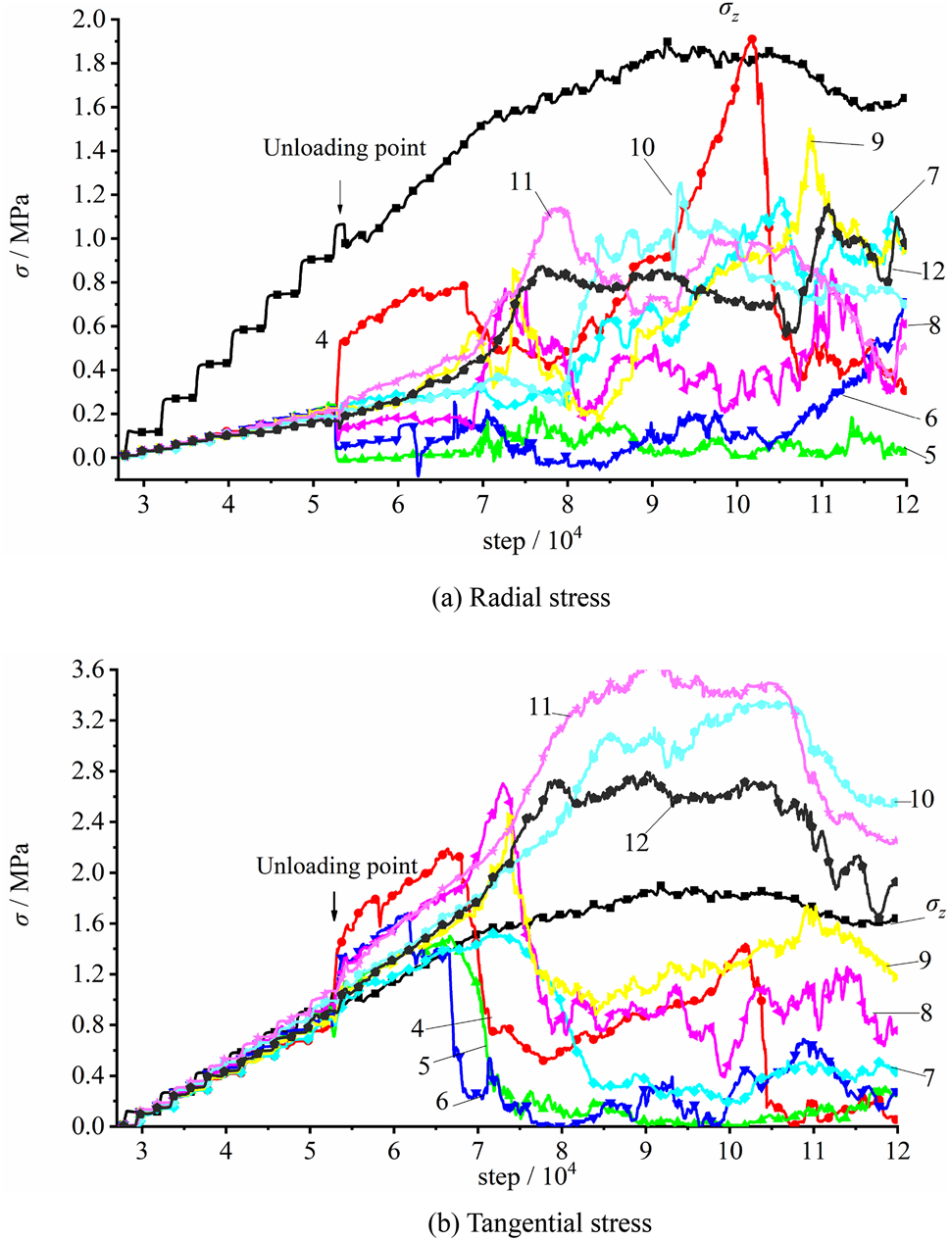
The stress variation of the measuring element at the vertical sidewall during excavation with imposed vertical stress of 0.90 MPa.

The stress variation of the measuring element at the vertical sidewall with the initial rock stress of 60% σ_zmax_, namely 0.90 MPa, is plotted in Fig. 26. During the process of applying the initial rock stress, the stress of each measuring element grew linearly with the load imposed on the top surface of the model. The tangential stress σ_θ_ was equal to the imposed vertical stress σ_z_, and the radial stress σ_r_ was comparably small, with σ_θ_: σ_z_: σ_θ_ = 0.21: 1.0: 1.0. When the imposed stress grew to 0.90 MPa, tunnel excavation initiated. The tangential stresses of E4 and E6 rose up rapidly (σ_θ_^4^ = 1.55 MPa and σ_θ_^6^ = 1.30 MPa) while the radial stresses of E5 and E6 declined which indicated during the excavation under lower vertical stress, the radial stress declined, the tangential stress grew and the tunnel as a whole generally remained stable.

During the loading process after excavation, the tangential stresses of E4 and E6 gradually grew. As the imposed load reached 1.200 MPa, the tangential stress of E6 peaked at 1.52 MPa and then rapidly declined. Similarly, with the vertical load of 1.383 MPa, the tangential stress of E4 reached its maximum value 2.19 MPa and dropped quickly afterwards. This illustrated that the surrounding rock of the tunnel free face failed and was no longer intact. It should also be noted that the stresses of E7 − E9 still rose up rapidly. With vertical load of about 1.583 MPa, the stresses of E7 − E9 reached the maximum value one after another (σ_θ_^7^ = 1.51 MPa, σ_θ_^8^ = 2.70 MPa and σ_θ_^9^ = 2.45 MPa), and they also encountered fast reduction subsequently, which suggested an enlarged loosened zone existed in the surrounding rock. As the imposed load continued growing, the load capacity of model firstly reached its peak and then fell, while the measuring spot located in the deeper surrounding rock encountered its peak stress successively, and the failed and loosened zone of the surrounding rock further expanded.

**Figure 27:**
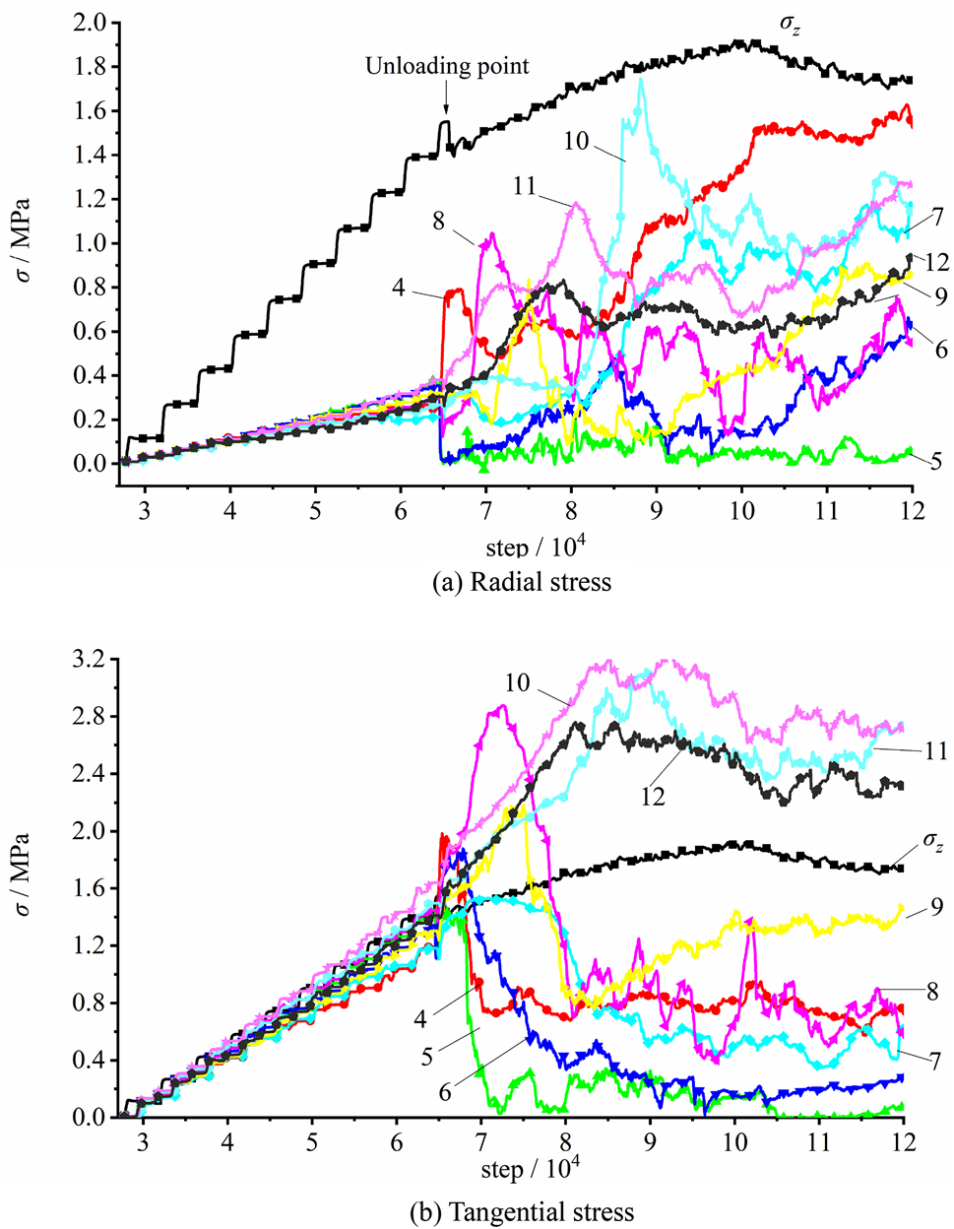
The stress variation of the measuring element at the vertical sidewall during excavation with imposed vertical stress of 1.500 MPa.

The stress variation of the measuring element at the vertical sidewall during excavation unloading with the initial vertical rock stress of 100% σ_zmax_, namely 1.500 MPa, is illustrated in Fig. 27. As the imposed vertical stress grew to the set value, the stress of each measuring element increased linearly. The tangential stress σ_θ_ was gin good agreement with the vertical stress imposed on the top surface of the model σ_z_, and the radial stress σ_r_ was lower than the former two, with σ_r_: σ_z_: σ_θ_ = 0.21: 1.0: 1.0. The excavation started after the vertical stress reaching the given value. The radial stress of E4 rapidly grew to its peak value of 0.79 MPa then dropped, and those of the E5 and E6 quickly reduced to zero. At the same time, the tangential stresses of E4 − E6 all rapidly climbed up to their maximum values (σ_θ_^4^ = 1.96 MPa, σ_θ_^5^ = 1.46 MPa and σ_θ_^6^ = 1.84 MPa) then dived instantly, which suggested that the surrounding rock of the free face was quickly damaged and loosened due to the higher initial rock stress. During the early unloading, E7 − E9 did not appear failure. Then, after 8000 times of iteration, the imposed vertical load reached 1.550 MPa, and the stresses of E7 − E9 climbed up to their peaks. The peak stresses of E7 were σ_r_ = 0.30 MPa and σ_θ_ = 1.54 MPa; for E8, σ_r_ = 1.05 MPa and σ_θ_ = 2.88 MPa; for E9, σ_r_ = 0.83 MPa and σ_θ_ = 2.17 MPa. The stresses of E7 − E9 then declined with the continuing loading correspondingly. This indicated the expansion of the failed and loosened zone of the surrounding rock. As iteration continued, the measuring spot deeper inside the model successively encountered its peak stress, with the loosened zone of the surrounding rock constantly enlarging.

**Figure 28:**
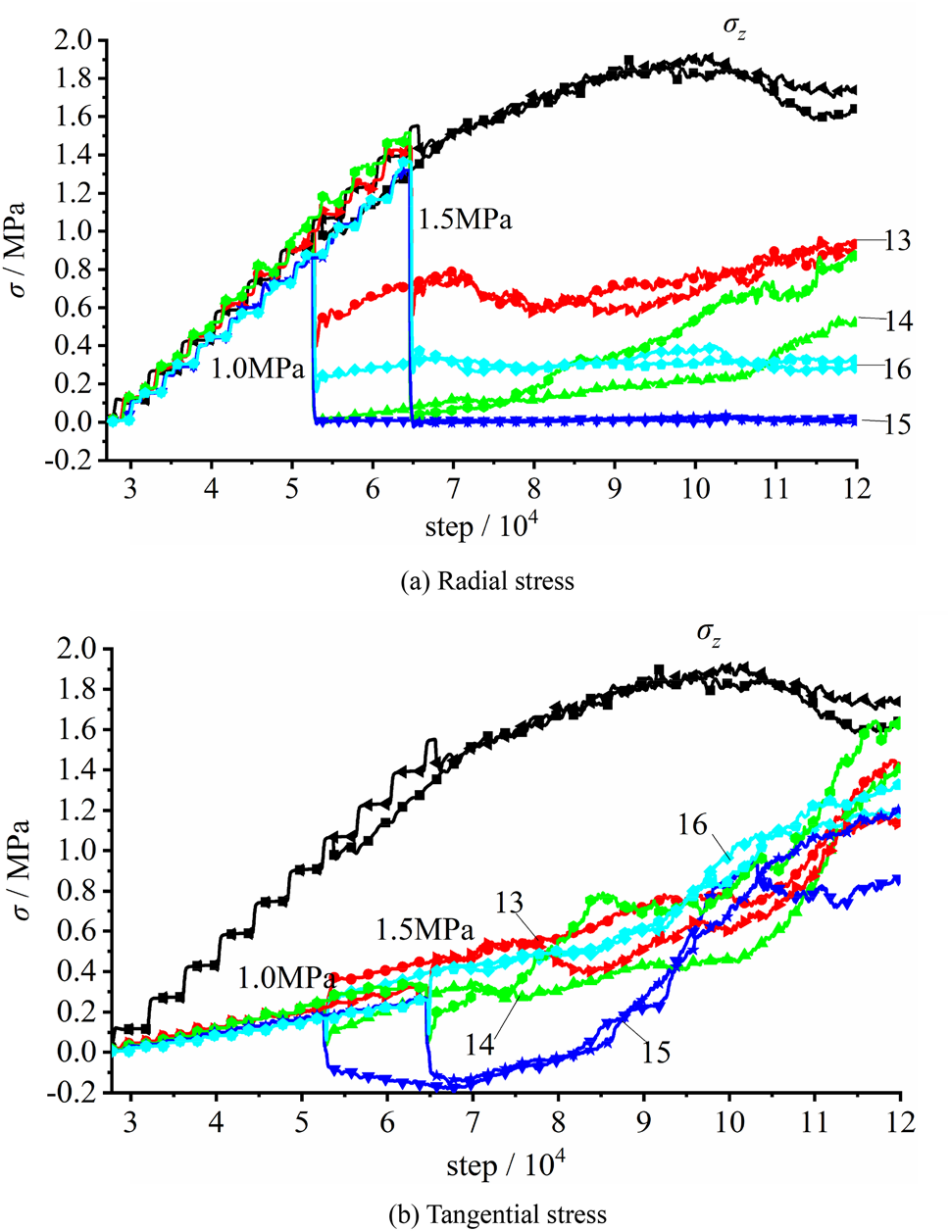
The stress variation of the measuring element at the arch crown and bottom during excavation unloading.

Figure 28 shows the stress variations of the measuring elements at the arch crown and bottom during the excavation unloading with imposed vertical stresses of 0.900 MPa and 1.500 MPa, respectively, which illustrated that the stress variation tendencies were similar under varied initial vertical stresses. The stress of each measuring element increased linearly with the growing vertical stress, as the latter one gradually increased to the set value. The tangential stress σ_θ_ was equal to the imposed vertical stress σ_z_ and the radial stress σ_r_ was about 21% of σ_z_.

Tunnel excavation started since the imposed vertical stress reached the set value. In terms of the radial stress, they rapidly declined to low values at E13 − E16. The σ_r_ of E13 reduced to 0.66 MPa or so, while that of E16 reduced to about 0.30 MPa. Moreover, the stresses of E13 and E16 inside the surrounding rock were higher than those of E14 and E15 on the tunnel internal surface. The radial stress basically remained constant afterwards. As for the tangential stress, the changes were quite similar for E14 and E15 yet the stresses of E13 and E16 to some extent rose up. The tangential stress of E5 was in tensile state which grew to its absolute peak value of 0.18 MPa, with the increasing stress imposed on the top surface of the model. The tensile stress then gradually declined and transformed into the pressured state, as the imposed stress reached the peak load. This revealed that the bottom of the model did not always in tensile state during loading which was consistent to the result of the physical model testing. E13, E14 and E16 were all in the pressured state along their tangential directions and their stresses grew to 30% σ_z_. Apparent inflection points could be observed in the stress variation of each measuring element, after which the stress rapidly grew.

During the excavation − induced unloading, the growth or reduction magnitudes of stress of measuring elements at the arch crown and bottom with the imposed vertical stress of 1.5 MPa all surpassed those with imposed vertical stress of 0.90 MPa. Nevertheless, no obvious difference was found between the two cases during the loading process after the completion of excavation. Compared with the loading testing, the stress variation of the measuring element was to some extent accelerated.

## Conclusions

This paper studies the failure mechanism of the tunnel during the loading and unloading processes, on the basis of the comparison between physical model testing and discrete element method − based simulation. The conclusions are drawn as follows:

- (1) The results of physical testing and numerical simulation during loading were consistent and both indicated that the arch bottom was in tensile state, while the arch crown was in pressured state. The tangential stress of the arch bottom and the radial stress of the sidewall were tensile, which led to the initiation and propagation of fractures around surrounding rock. In addition, the failure of surrounding rock mostly occurred at the two vertical sidewalls because shear fractures propagated obliquely upward and downward at the foot of the wall and the spandrel, respectively, which cut it into a wedge. After the sliding plane of the wedge moved inwards and the width of fractures grew, several tensile rupture planes were formed which resulted in layer-by-layer collapse of the sidewall. The failure pattern transformed from the tensile fracture of the arch bottom with lower load to the collapse of the damaged and extruded sidewall under the combined shear and tensile deformation with high load.
- (2) The unloading testing analyzed two cases respectively with imposed vertical stress of 60% and 100% determined peak load (strength), σ_zmax_. The failure mode presented in the numerical simulation was in good agreement with that in model testing. Fractures were only found in a small zone around the vertical sidewall, with the imposed load of 60% σ_zmax_. Yet, the surrounding rock of the tunnel was damaged and fractured, and the failure mostly occurred at the sidewall when exerted load was 100% σ_zmax_. In both cases, the failure pattern was found to be the collapse of the V − shaped damaged and extruded sidewall under the joint effects of the shear failure of the sidewall wedge and the vertical tensile failure.
- (3) The comparison between loading and unloading processes illustrated that certain tensile strain generated at the arch bottom under these two situations. However, the strain growth rates at the vertical sidewall and the arch haunch during unloading exceeded those in the loading process, which meant that the extrusion of the surrounding rock towards the free face as well as the rock failure during unloading developed faster. The surrounding rock of the vertical sidewall happened overall collapse with high integrity of rock mass in the case of loading. As for unloading, the surrounding rock of the two sidewalls that extruded towards the free face broke in the middle position due to the deterred sliding of the lower part of the extrusion body, and the integrity of rock mass was poor. Therefore, the rock failure was much more severe during the unloading process.
